# FGF15 promotes neurogenesis and opposes FGF8 function during neocortical development

**DOI:** 10.1186/1749-8104-3-17

**Published:** 2008-07-14

**Authors:** Ugo Borello, Inma Cobos, Jason E Long, Cornelis Murre, John LR Rubenstein

**Affiliations:** 1 Nina Ireland Laboratory of Developmental Neurobiology, Department of Psychiatry, University of California, San Francisco, CA 94143, USA; 2 Department of Biology, University of California, San Diego, CA 92093, USA; 3 ICREA and Department of Cell Biology, University of Barcelona. Avda. Diagonal, 08028 Barcelona, Spain; 4 Genentech, Inc., DNA Way, South San Francisco, CA 94080, USA

## Abstract

**Background:**

Growth, differentiation and regional specification of telencephalic domains, such as the cerebral cortex, are regulated by the interplay of secreted proteins produced by patterning centers and signal transduction systems deployed in the surrounding neuroepithelium. Among other signaling molecules, members of the fibroblast growth factor (FGF) family have a prominent role in regulating growth, differentiation and regional specification. In the mouse telencephalon the rostral patterning center expresses members of the *Fgf *family (*Fgf8*, *Fgf15*, *Fgf17*, *Fgf18*). FGF8 and FGF17 signaling have major roles in specification and morphogenesis of the rostroventral telencephalon, whereas the functions of FGF15 and FGF18 in the rostral patterning center have not been established.

**Results:**

Using *Fgf15*^-/- ^mutant mice, we provide evidence that FGF15 suppresses proliferation, and that it promotes differentiation, expression of *CoupTF1 *and caudoventral fate; thus, reducing *Fgf15 *and *Fgf8 *dosage have opposite effects. Furthermore, we show that FGF15 and FGF8 differentially phosphorylate ERK (p42/44), AKT and S6 in cultures of embryonic cortex. Finally, we show that FGF15 inhibits proliferation in cortical cultures.

**Conclusion:**

FGF15 and FGF8 have distinct signaling properties, and opposite effects on neocortical patterning and differentiation; FGF15 promotes *CoupTF1 *expression, represses proliferation and promotes neural differentiation.

## Background

Regional specification, growth and differentiation of telencephalic subdivisions, such as the cerebral cortex, are regulated by the interplay of secreted proteins produced by patterning centers and signal transduction systems deployed in the surrounding neuroepithelium. The dorsal telencephalic patterning center is the source for bone morphogenetic proteins (BMPs) and Wnts that regulate development of dorsal and caudal parts of the telencephalon, including the choroid plexus and hippocampus [1-3]. A putative ventral patterning center expresses sonic hedgehog (SHH); dorsoventral patterning of the telencephalon is controlled through the GLI3 repressor of the SHH pathway [4-6]. An additional putative patterning center at the cortical-subcortical junction (pallium-subpallium boundary (PSB)) expresses multiple signaling molecules, such as *Tgfα*, *Neuregulin 1 *and *3*, *Fgf7*, the putative Wnt antagonist *Sfrp2 *[7,8] and *Fgf15 *(this paper). Finally, the rostral patterning center expresses members of the *Fgf *family (*Fgf8*, *Fgf15*, *Fgf17*, *Fgf18*) [9-13].

The mammalian fibroblast growth factor (FGF) family consists at least 22 members whose functions range from specifying regional and cell fate to promoting proliferation, differentiation and survival [14]. FGF ligand binding to their cognate tyrosine kinase receptors (FGFR1-4) activates two major phosphorylation cascades: the Ras/mitogen activated protein kinase (MAPK) and the phosphatidylinositol 3-kinase/AKT pathways [14].

FGF signaling is implicated in early steps of neural induction [14]. As the neural plate matures, its rostral margin, known as the anterior neural ridge, expresses at least three members of the *Fgf *family, *Fgf8*, *Fgf17 *and *Fgf18 *[9-12]. Following neurulation, these genes continue to be expressed in the rostral midline of the forebrain. *Fgf15 *expression is also prominent in the early rostral forebrain [15-17]. Within the rostral patterning center, *Fgf8 *and *Fgf18 *are expressed in its core, whereas *Fgf17 *expression extends dorsally [18] and *Fgf15 *extends ventrally [13,16,17,19]. *Fgf15 *is also expressed at the PSB and in the caudal-most region of the ventral pallium [16,17,19,20].

FGF8 and FGF17 signaling have major roles in specification and morphogenesis of the rostroventral telencephalon. Within the cortex, FGF8 is essential for producing most of the frontal cortex [17,21,22], whereas FGF17 is essential for the development of the dorsomedial frontal cortex [17,18]. The functions of FGF15 and FGF18 in the rostral patterning center have not been established, although *Fgf18 *expression in the cortical plate is implicated in laminar patterning and neuronal migration [23].

FGF8 promotes proliferation and survival of telencephalic progenitor cells, and specifies rostral telencephalic fate through positively regulating expression of several transcription factors, including *Erm*, *Er81*, *Foxg1*, *Nkx2.1*, *Pea3 *and *Sp8 *[16,17,19,22,24,25]. In addition, FGF8 represses caudal telencephalic fate through reducing the expression of *CoupTF1*, *Emx2 *and *Wnt8b *[12,21,22,25-27]. *Fgf17 *expression is genetically downstream of *Fgf8 *(this paper) and it is important in local patterning within the rostral cortex [17,18].

FGF8, FGF17 and FGF18 belong to the same subfamily based on their amino acid sequence, whereas FGF15, known as FGF19 in humans, chickens and zebrafish, is part of a distinct subfamily [28,29].

Here we explore the functions of FGF15 in neocortical development. Previous loss of function studies in the mouse established its function in the development of the gall bladder, inner ear, and cardiac outflow tract [30-33]. The function of the zebrafish *Fgf15 *homologue (*Fgf19*) has been assessed in the developing brain using antisense (morpholino) oligonucleotides [34]. That study provided evidence that *Fgf15*(*19*) promotes proliferation, similar to known functions of other *Fgf*s. On the other hand, our analysis of *Fgf15*^-/- ^mutant mice, and primary cortical cultures treated with recombinant FGF15, provides evidence that FGF15 suppresses proliferation and promotes differentiation in the developing telencephalon. Furthermore, we demonstrate that *Fgf15 *promotes expression of *CoupTF1*, a transcription factor that represses proliferation, and promotes differentiation and caudoventral fate. Thus, *in vivo *reduction of *Fgf15 *dosage has the opposite effect of reducing *Fgf8 *dosage. Finally, in primary cortical cultures, we demonstrate that FGF15 and FGF8 have distinct effects on phosphorylation of ERK (p42/44), AKT, and S6, providing a link between signaling differences and the distinct cellular effects of these proteins.

## Results

### *Fgf15 *expression is repressed by FGF8 and is promoted by SHH

**Figure 1:**
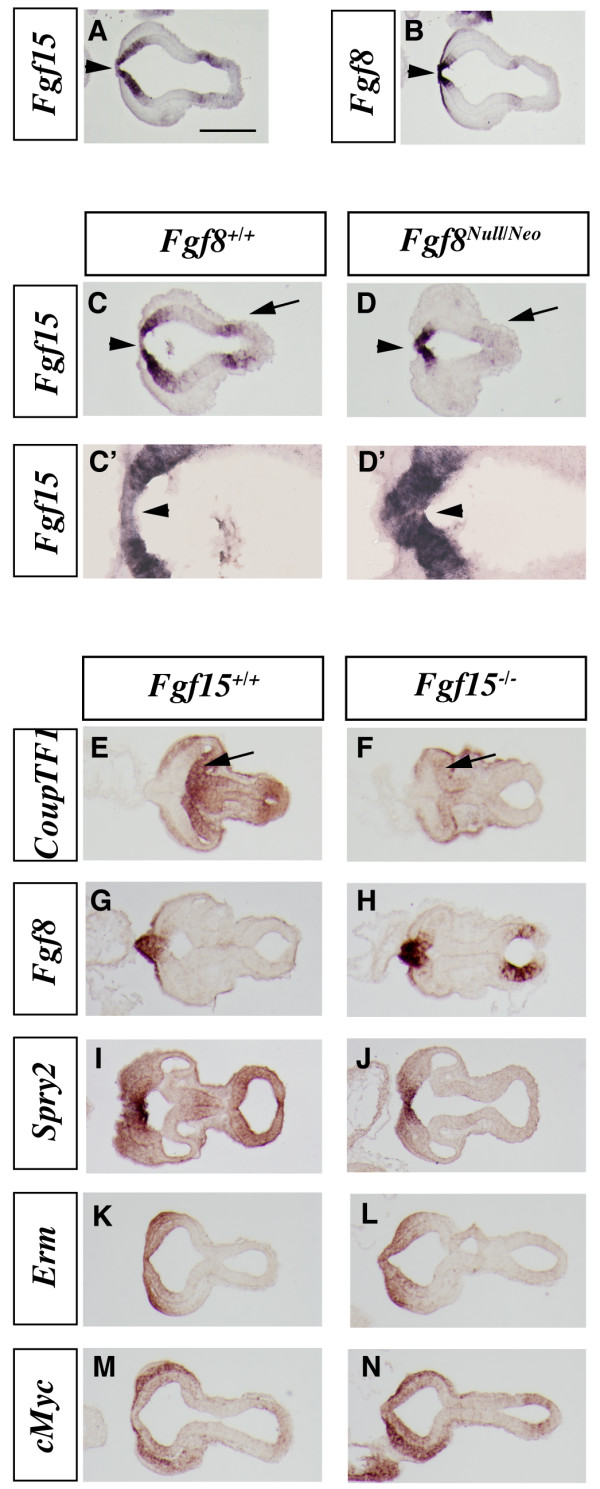
***In situ*****RNA hybridization on horizontal sections in wild-type,*****Fgf8***^***Null***/***Neo***^**and *****Fgf15***^**-/-**^**E9.5 embryos.** (a, b) *Fgf15 *(a) and *Fgf8 *(b) expression in adjacent sections of a wild-type embryo. (c-d') *Fgf15 *expression in wild-type (c) and *Fgf8*^*Null*/*Neo *^embryos (d). Higher magnifications of (c, d) are shown in (c', d'). (e-n) Expression analysis in wild-type and *Fgf15 *mutant embryos showing adjacent sections of *CoupTF1 *(e, f), *Fgf8 *(g, h), *Spry2 *(i, j), *Erm *(k, l) and *cMyc *(m, n). Arrowheads in (a, b): rostral midline that is *Fgf8*^+ ^and *Fgf15*^-^; the midline becomes *Fgf15*^+ ^in *Fgf8*^*Null*/*Neo *^(d, d'). Arrow in (d): loss of *Fgf15 *expression in the midbrain of the *Fgf8*^*Null*/*Neo *^mutant. Arrows in (e, f): reduced *CoupTF1*in *Fgf15*^-/-^. Bar in (a) is 200 μm.

To assess the role of FGF15 in cortex development, we extended the analysis of *Fgf15 *expression in the developing telencephalon by *in situ *RNA hybridization, to complement previous reports [16,17,19]. At embryonic day 9.5 (E9.5), *Fgf15 *was expressed in the anterior forebrain neuroepithelium (Figure 1a), but was excluded from the anterior dorsal midline where *Fgf8 *was expressed (Figure 1a, b, arrowhead). At E12.5, *Fgf15 *was strongly expressed in the septum (Additional file 1g, arrow), preoptic area (Additional file 1h, arrow), and weakly expressed in the neuroepithelium of the PSB (Additional file 1g, arrowhead). In addition, *Fgf15 *was strongly expressed in the caudal ganglionic eminence (CGE; Additional file 1j, arrow).

The overlapping and complementary expression of *Fgf15 *and *Fgf8 *in the dorsal midline suggests regulatory interactions between these factors. Gain-of-function experiments by Gimeno and Martinez [16] demonstrated that FGF8 protein can induce *Fgf15 *expression in the mesencephalon and prosencephalon. Thus, we analyzed the expression of *Fgf15 *in severely hypomorphic *Fgf8 *mutants (*Fgf8*^*Null*/*Neo*^) [22]. At E9.5, *Fgf15 *expression was strongly reduced at the midbrain/hindbrain boundary (Figure 1c, d, arrows), consistent with Gimeno and Martinez [16]. However, in the rostral forebrain, *Fgf15 *expression remained strong, and it was ectopically expressed in the anterior midline (Figure 1c, d, c', d', arrowheads). At E12.5, *Fgf15 *expression remained strong in the dysmorphic septal area and appeared to be increased in the CGE (Additional file 1e–j'). Thus, while *Fgf15 *is positively regulated by FGF8 in the midbrain, it is repressed by FGF8 in the prosencephalic midline.

There is evidence that SHH promotes *Fgf15 *expression in the diencephalon [35] and in the cortex [6,20]. Our analysis of the *Shh*^-/- ^mutant extended these findings by demonstrating that *Fgf15 *expression in the rostral telencephalon requires SHH function (Additional file 2). Thus, while *Fgf15 *expression in the rostral telencephalon requires SHH, it remains strong in the severe *Fgf8 *hypomorph, despite the lack of telencephalic *Shh *expression in these mutants [22]. In addition, *Gli3*, a repressor of the SHH signal, may to be over-expressed in the *Fgf15 *mutants at E12.5 (Additional file 2q, q'), suggesting a positive feedback between FGF15 and SHH.

### Opposite effects of FGF15 and FGF8 on patterning gene expression in the cortical primordium

**Figure 2:**
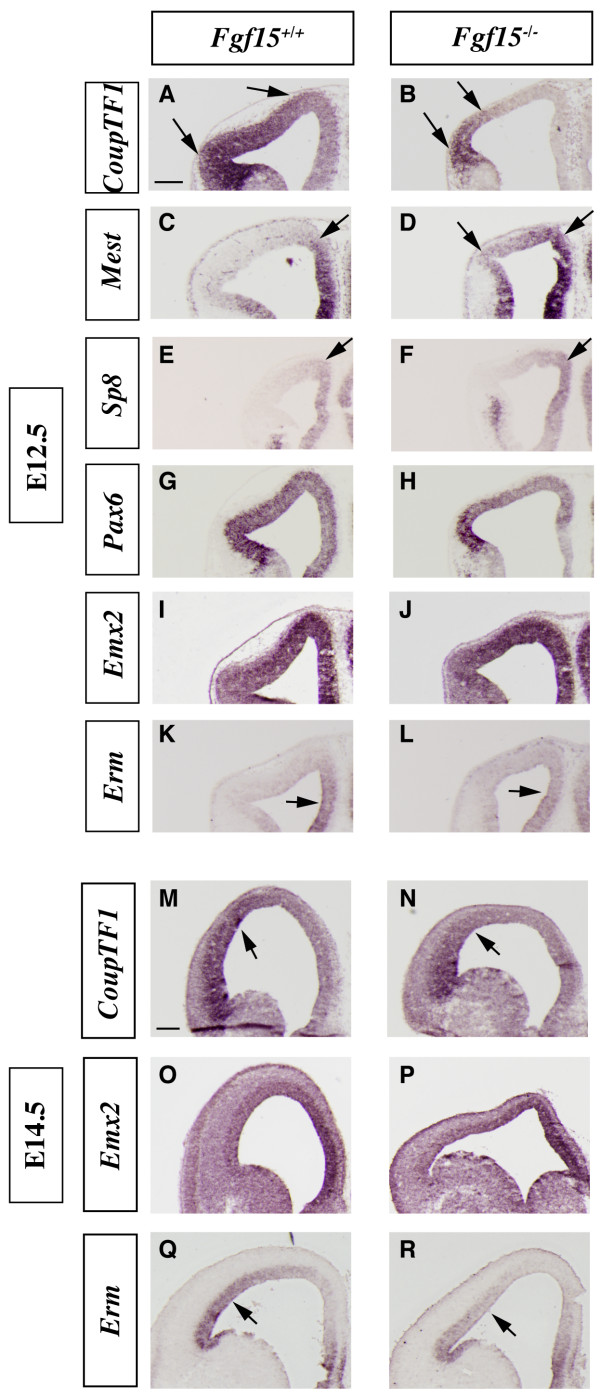
**Analysis of the cortical patterning defects in the *Fgf15 *mutants.** (a-r) *In situ *RNA hybridization on coronal sections of *Fgf15*^+/+ ^(left column) and *Fgf15*^-/- ^(right column) embryos at E12.5 (a-l) and E14.5 (m-r). *CoupTF1 *(a, b, m, n), *Mest *(c, d), *Sp8 *(e, f), *Pax6 *(g, h), *Emx2 *(i, j, o, p) and *Erm *(k, l, q, r). Arrows highlight the changes in the extent and/or intensity of expression. Bar in (a, m) is 200 μm.

**Figure 3:**
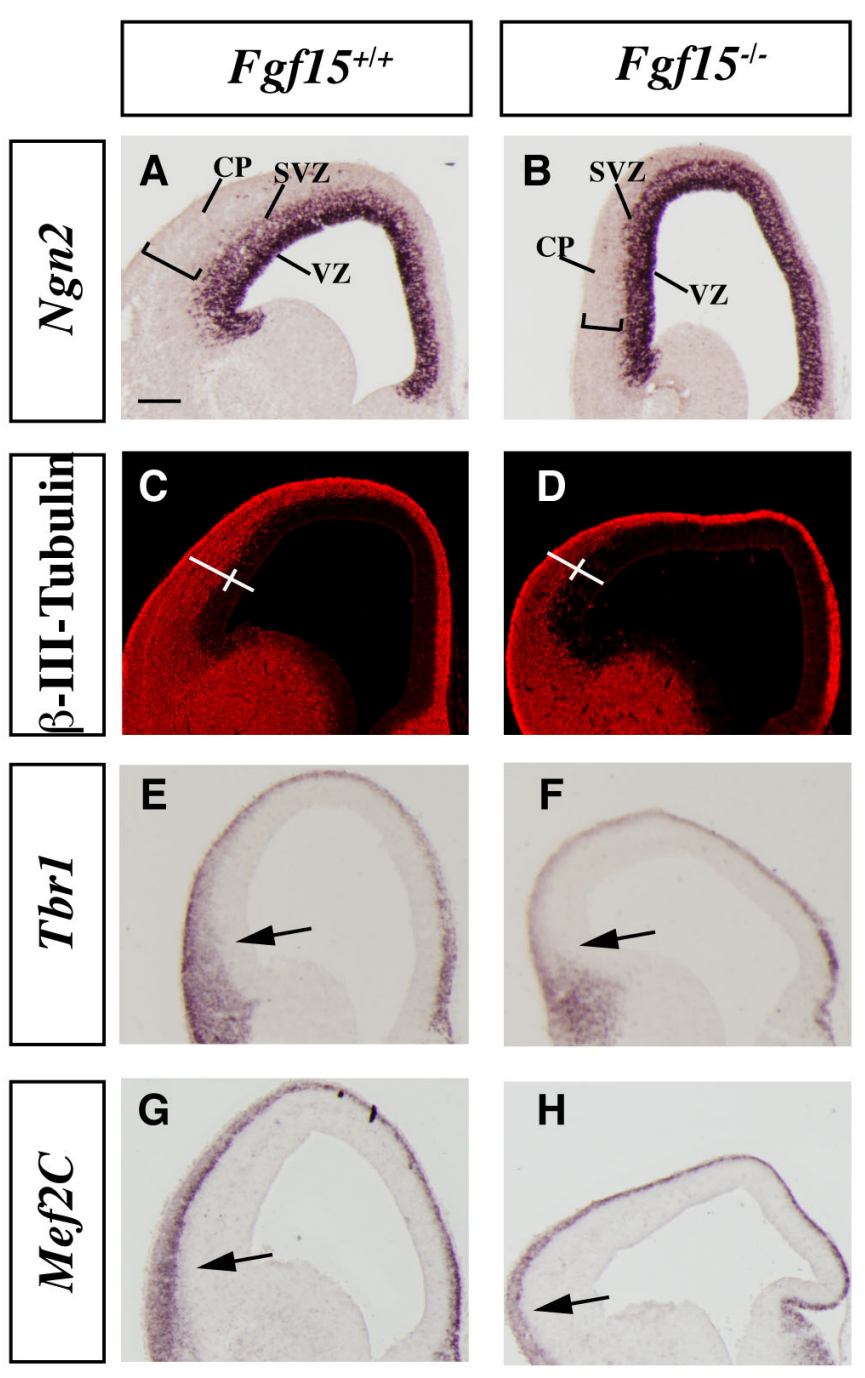
**Analysis of the cortical neurogenesis defects in the *Fgf15 *mutants.** (a-h) *In situ *RNA hybridization (a, b, e-h) and immunofluorescence (c, d) on coronal sections of *Fgf15*^+/+ ^(left column) and *Fgf15*^-/- ^(right column) embryos at E14.5. *Ngn2 *(a, b), β-III-Tubulin (c, d), *Tbr1 *(e, f), *Mef2C *(g, h). Arrows point out the reduced thickness of the cortical plate expression of *Tbr1 *and *Mef2C*. Brackets in (a-d) highlight the reduced thickness of the cortical plate. CP, cortical plate; SVZ, subventricular zone; VZ, ventricular zone. Bar in (a) is 200 μm.

To determine FGF15's functions in forebrain development we analyzed the *Fgf15 *null (*Fgf15*^-/-^) mutant mouse [32], and focused on the cortical phenotype. Histological analysis showed that the mutant cortex was thinner than the wild type (Figures 2 and 3; quantification below). The gross morphology of *Fgf15 *mutant embryos is similar to the wild-type cohort (Additional file 3).

We started the analysis at E9.5, soon after neurulation has generated the telencephalic vesicles, by studying mRNA expression of genes known to have important roles in cortical development or known to be markers of FGF signaling.

*CouTF1 *promotes caudoventral cortical identity [36-38]. While reducing *Fgf8 *dosage increased *CoupTF1 *expression [21,22], we observed that loss of *Fgf15 *strongly reduced its expression at E9.5 (Figure 1e, f). Subtle changes in expression are also observed, including increased *Fgf8 *expression (Figure 1g, h); however, establishing these changes would require more quantitative methods. On the contrary, we did not observe a major effect on the expression of two genes that are positively regulated by FGF signaling, *Spry2 *(Figure 1i, j) and *Erm *(Figure 1k, l). Expression of *cMyc*, a gene downstream of the Ras/MAPK signaling [39,40], may be slightly increased (Figure 1m, n).

Next, we interrogated FGF15 function by analyzing *CoupTF1*, *Mest*, *Sp8*, *Pax6*, *Emx2 *and *Erm *expression in the cortical ventricular and mantle zones at E12.5 and E14.5. As at E9.5, *CoupTF1 *expression was strongly reduced (Figure 2a, b and 2m, n), in contrast with its increase in *Fgf8 *mutants [21,22]. *Fgf15 *and *Fgf8 *mutants also showed the opposite effects on *Mest *and *Sp8 *expression (Figure 2c, d and 2e, f, respectively) [22,41].

On the other hand, *Pax6*, *Erm *and *Emx2 *showed subtle changes in the *Fgf15 *mutant. *Pax6 *cortical ventricular zone expression showed a modest decrease at E12.5 (Figure 2g, h) as well as *Erm *at E12.5 and E14.5 (Figure 2k, l and 2q, r). *Emx2 *expression may be increased in the dorsomedial cortical ventricular zone at E12.5 and E14.5 (Figure 2i, j and 2o, p). At this stage *Fgf15 *expression in the rostral patterning center does not appear to overlap with the cortical expression of the gene analyzed here. The changes in cortical expression could, however, result from the earlier patterning changes observed at E9.5 (Figure 1) and through diffusion of FGF15.

Zebrafish treated with morpholinos to *Fgf19 *(the name of zebrafish *Fgf15*) exhibit loss of subpallial molecular features [34]. We, however, did not detect a strong subpallial phenotype in the mouse *Fgf15 *mutant, although there was a suggestion of reduced *GAD67*,*Nkx2.1 *and *Pax6 *expression in the mantle zone of the ventral septum and piriform cortex (Additional file 4). There was, however, a clear reduction in the number of *GAD67*^+^cortical interneurons in the dorsomedial cortex (Additional file 4a, b). *Reelin *expression in this region appeared normal showing that differentiation of the marginal zone was not grossly disrupted (Additional file 4e, f).

In summary, reduced expression of *Fgf15 *and *Fgf8 *has opposite effects on the expression of several transcription factors that have prominent functions in regulating cortical regional fate, proliferation and differentiation. This was particularly clear for *CoupTF1*, which promotes caudoventral cortical fate [37,38], and which represses proliferation and promotes differentiation [38].

### FGF15 promotes maturation of the cortical neural precursors in the ventricular and subventricular zones

The cortex in the *Fgf15 *mutants was thinner than the wild type at E14.5 (Figures 2 and 3), suggesting that FGF15 may regulate the balance between proliferation and differentiation. To explore this, we analyzed markers of differentiation and indices of proliferation.

*Neurogenin2 *(*Ngn2*) expression marks progenitor cells in the cortical ventricular zone (VZ) and subventricular zone (SVZ) and is largely excluded from the postmitotic neurons of the cortical plate [42] (Figure 3a). *Fgf15 *mutants maintained strong *Ngn2 *expression in the VZ, while its expression in the SVZ seemed to be reduced; however, the *Ngn2 *negative area was much thinner (Figure 3a, b), suggesting reduced cortical plate thickness. To confirm this observation, we studied expression of β-III-Tubulin, a marker of differentiating neurons. Indeed, the width of the cortical postmitotic zone was greatly reduced in the *Fgf15 *mutant (50% in ventral regions and 45% in dorsal regions; Figure 3c, d), while the width of the VZ was larger in the mutants (30% in ventral regions and 25% in dorsal regions; Figure 3c, d). The reduced thickness of the cortical plate was confirmed by analyzing expression of markers of early born cortical projection neurons, *Tbr1 *and *Mef2C *(Figure 3e–h). *Tbr1 *and *Mef2C *expressions were reduced in the cortical plate of the *Fgf15 *mutants (Figure 3e, f, g, h). Thus, *Fgf15 *mutants show a defect in the growth of the cortical plate, perhaps due to defect in neurogenesis.

**Figure 4:**
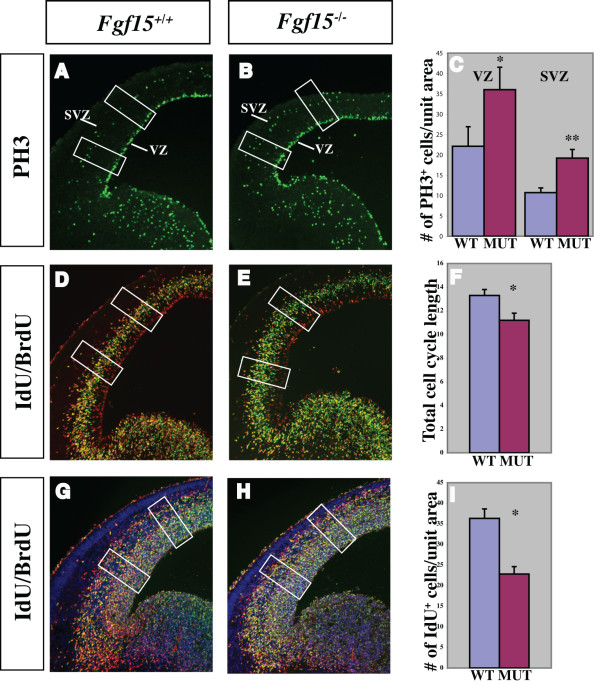
**Analysis of proliferation and cell cycle in the *Fgf15 *mutants.** (a-i) Comparison of the rate of proliferation of the neuronal progenitors at E14.5 in coronal hemisections from *Fgf15*^+/+ ^(left column) and *Fgf15*^-/- ^(right column) embryos. The number of neuronal progenitors undergoing mitosis was evaluated by PH3^+ ^immunofluorescence (a, b); quantification of the PH3^+ ^cells in the VZ and SVZ is shown in (c) (n = 3; *p *= 0.0019, Student's *t*-test, indicated by * and **; see Materials and methods). The analysis of the cell cycle length (d, e) (quantification in (f)) and of the number of neuronal progenitors exiting the cell cycle (Q fraction) (f, g) (quantification in (i)) were performed at E14.5 by double labeling with IdU and BrdU (n = 3; *p *= 0.001, Student's *t*-test, indicated by *; see Materials and methods). The rectangles in a-b, d-e, g-h indicate the sampled bins. The error bars indicate the standard deviations. MUT, mutant; SVZ, subventricular zone; VZ, ventricular zone; WT, wild type.

We explored the hypothesis that the cortical plate defect is caused because FGF15 may promote the switch between proliferation and differentiation. Thus, we assessed the mitotic index by immunostaining with phospho-histone H3 (PH3), a marker for M phase of the cell cycle. At E14.5, we observed a 37% increase in the density of PH3^+ ^cells in the cortical VZ and a 42% increase in the SVZ (Figure 4a–c). This was already evident at E12.5 when the increase was 20% (Additional file 5a–c).

The rate of proliferation of the cortical precursor cells is linked to the length of the cell cycle; in addition, the fraction of cells in a given phase of the cell cycle is directly proportional to the length of that specific phase, relative to the total length of the cell cycle [43,44]. Sequentially exposing proliferating cells to iodo-deoxyuridine (IdU) and bromo-deoxyuridine (BrdU) allows one to differentiate between defined populations of proliferating cells and then to calculate the cell cycle length (see Materials and methods). We compared the length of the cell cycle in E12.5 and E14.5 wild type and *Fgf15 *mutants using IdU/BrdU double labeling [45] (Figure 4d–f; Additional file 5d–f). Cell cycle length in the mutants was reduced to 11 hours, compared to the 13.5 hours of the wild type at E14.5 (Figure 4d–f), and 10 hours, compared to the 11 hours of the wild type at E12.5 (Additional file 5d–f).

Next, we analyzed the fraction of neural precursor cells exiting the cell cycle to become neurons (Q fraction) at E14.5. To identify cells exiting the cell cycle, we used differential labeling by IdU and BrdU [45]. The IdU/BrdU double labeling method allows one to distinguish between cells that are proliferating from cells that exit the cell cycle (see Materials and methods). *Fgf15 *mutants showed a 38% reduction in Q fraction (cells exiting the cell cycle) (Figure 4g–i).

Overall, these data provide evidence that reducing the dosage of *Fgf15 *reduces cell cycle length and cell cycle exit. Thus, FGF15 induces neurogenesis and this observation contrasts with the typical function of FGFs in promoting proliferation and maintenance of the neural progenitor state [14].

### Analysis of the Wnt and the retinoic acid signaling pathways in the *Fgf15 *mutants

**Figure 5:**
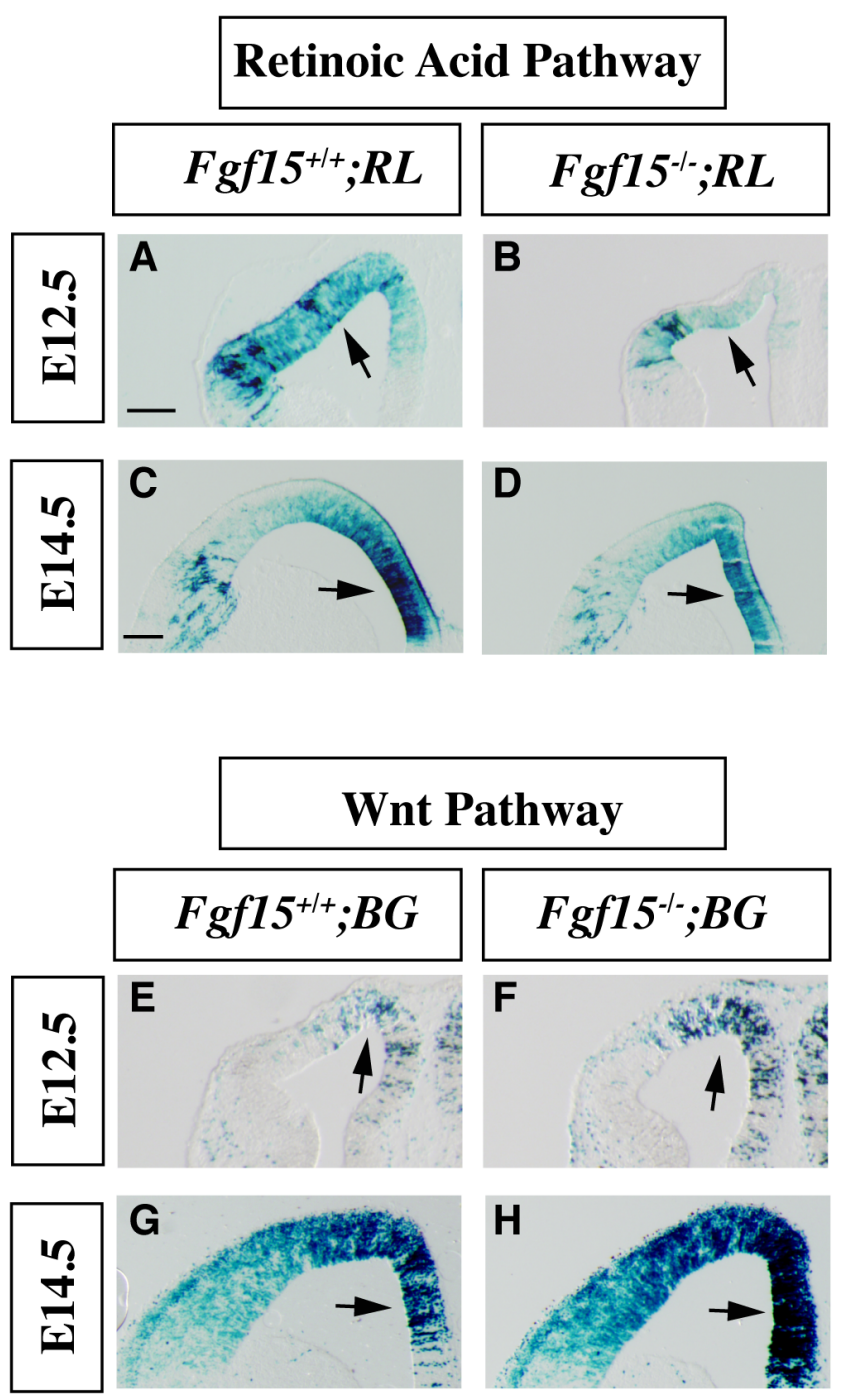
**Activation of the Wnt/β-catenin and retinoic acid signaling pathways in the *Fgf15 *mutants.(a-d) **Retinoic acid pathway activation was determined at E12.5 (a, b) and E14.5 (c, d) by β-galactosidase staining of coronal sections of *Fgf15*^-/-^; RL^+ ^embryos (RL, *RARE LacZ*, retinoic acid reporter). **(e-h) **The Wnt/β-catenin pathway activation was revealed by β-galactosidase staining of coronal sections of *Fgf15*^-/-^; BG^+ ^(BG, *BATgal*, Wnt/β-catenin reporter) at E12.5 (e, f) and E14.5 (g, h). Arrows indicate the regions that show the largest changes in the extent and/or intensity of expression. Three separate cases were analyzed for each genotype. Bar in (a, c) is 200 μm.

To investigate how FGF15 regulates cell cycle and neurogenesis, we studied signaling pathways that regulate the switch between proliferation and neurogenesis in *Fgf15 *mutants. First, we investigated the retinoic acid pathway, which promotes neural differentiation [46]. We introduced an allele that expresses *LacZ *under the control of retinoic acid receptor enhancer elements [47] into the *Fgf15*^-/-^background. At E12.5 and E14.5, there was a strong decrease in β-galactosidase activity in the mutants (Figure 5a–d), suggesting reduced retinoic acid signaling, consistent with the decrease in neuronal differentiation (Figure 3).

Wnt signaling through the β-catenin pathway promotes cortical proliferation [2,48-50] and represses differentiation of cortical neural progenitors [51]. We introduced a Wnt/β-catenin *LacZ *reporter allele [52] into the *Fgf15*^-/- ^mutants and observed increased numbers of β-galactosidase^+ ^cells in their cortex at E12.5 and E14.5 (Figure 5e–h), consistent with an increase in Wnt signaling, and the increase in proliferation (Figure 4).

### Expression of FGF pathway effector genes in *Fgf15 *mutants

Towards understanding the mechanism of FGF15 function, we examined the effect of *Fgf15 *mutation on selected components of the FGF signal transduction pathway in the rostral telencephalon at E9.5, E12.5 and E14.5. We focused on expression of *Fgf receptors *(*Fgfrs*) and *Sprouty2 *(*Spry2*); *Sprouty *genes encode negative regulators of the FGF signaling pathway that are induced by FGF8 [24,53,54].

**Figure 6:**
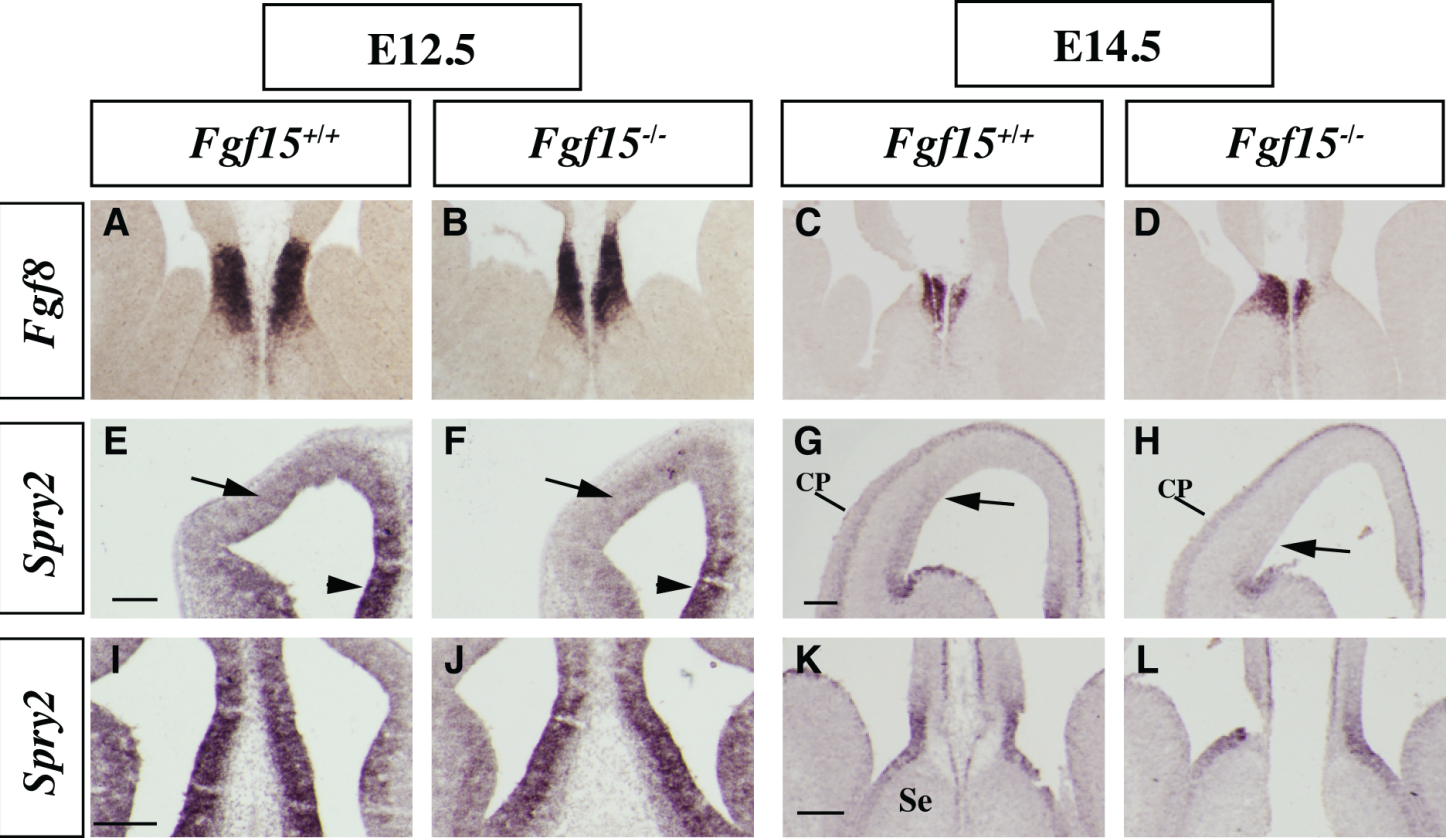
**Analysis of FGF signaling components in the *Fgf15 *mutants.** (a-l) *In situ *hybridization on coronal sections: *Fgf8 *at E12.5 (a, b) and E14.5 (c, d); *Spry2 *at E12.5 (e, f, i, j) and E14.5 (g, h, k, l). Arrows in (e-h) indicate the change in *Spry2 *gene expression in the lateral pallium; arrowheads in (e, f) indicate the change in the medial pallium. CP: cortical plate; Se: septum. Bars in (e, g, i, k) are 200 μm.

At E9.5 and E12.5, expression of *Fgf8 *and *Spry2 *in the rostral patterning center and the rostral midline (that is, anlage of the septum) appeared normal in the *Fgf15 *mutants (Figures 1g–j and 6a–l). At later stages, E12.5 and E14.5, *Spry2 *expression was reduced in the VZ of the ventrolateral cortex (Figure 6e–h, arrows) while its expression in the dorsomedial cortex was preserved (Figure 6e–f, arrowheads and Figure 6i–l).

Next we examined expression of *Fgfr1*-*4 *at E12.5 and E14.5. *Fgfr2 *showed the largest change in expression: it increased in the VZ, particularly in the dorsolateral cortex (Additional file 6e–h); in the cortical plates its expression was lost. *Fgfr1 *showed subtle changes in the VZ (a slight increase), but was reduced in the cortical plate and intermediate zone (Additional file 6a–d). *Fgfr3 *expression was reduced in the dorsal cortex at E14.5 (Additional file 6i–l, arrows).

FGF15 preferentially binds to FGFR4 *in vitro *[33,55-57]. We confirmed that *Fgfr4 *expression was not detectable in the E12.5 cortex (data not shown), as previously published [58]. However, we did observe its expression in the superficial cortical plate at E14.5; this expression domain was preserved, but thinner in the *Fgf15 *mutants (Additional file 6m–n). Thus, *Fgf15 *mutants showed varied effects on the expression of several FGF receptors in cortical progenitors and in the cortical plate.

### FGF15 and FGF8 differentially affect signaling pathways in cultured embryonic cortical cells

Toward establishing how FGF15 and FGF8 differentially affect cortical patterning proliferation and differentiation, we studied the effects of recombinant FGF15 and FGF8 proteins on dissociated E12.5 cortex; at this age most of the cortical cells are neuroepithelial progenitors.

Unlike mouse and human FGF2 and FGF8, which share 93% and 98% amino acid identity, mouse FGF15 shares only 51% identity with its human homolog FGF19 (data not shown) [28,29]. Thus, we compared the effect of purified mouse recombinant FGF15 protein with commercially available recombinant human FGF19 protein. Our results with FGF15 and FGF19 were indistinguishable (data not shown).

FGFs signal through two major pathways that when activated phosphorylate ERK (42/44) and the AKT [14,59]. These pathways can also activate other important kinases that regulate processes such as protein translation (S6 kinase) and Wnt signaling (GSK3 kinase).

**Figure 7:**
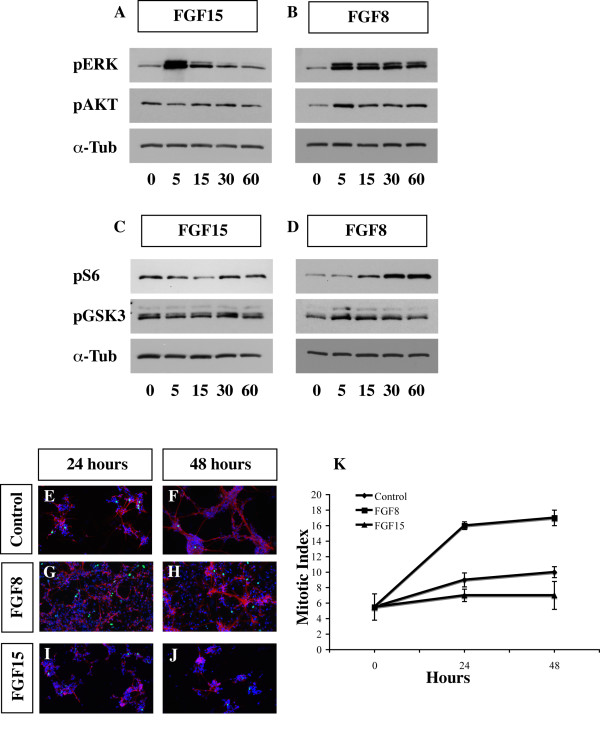
**Activation of the FGF15 and FGF8 downstream cytosolic effectors.** Comparison of phosphorylation levels of four proteins that are modified in response to FGF15 and FGF8. E12.5 primary cortical cultures were starved for 24 hours and then treated with recombinant FGF15 or FGF8. Cell lysates were analyzed after 0, 5, 15, 30 and 60 minutes by immunoblottingblotting to detect phosphorylated forms of pERK 42/44 (a, b, top panels), pAKT (a, b, middle panels) and pS6 (c, d, top panels), and pGSK3 (c, d, middle panels). The α-Tubulin (α-Tub) antibody (a-d, bottom panels) was used for normalization (these results are characteristic of what we observed the three times these experiments performed). The numbers under the bands indicate the fold-induction or reduction, with respect to T = 0, and are normalized with respect to the α-Tubulin level. (e-j) Immunofluorescence for β-III-Tubulin (red), phosphohistone-3 (PH3) (green) and Hoechst (blue) on E12.5 primary cortical cultures that were either not treated, treated with 50 ng/ml recombinant FGF8 or FGF15 for 24 and 48 hours (n = 4 for each experiment; *p *= 0.01, Student's *t*-test). (k) Graph showing the mitotic index calculated before starting the treatment (T0) and after 24 and 48 hours of treatment with recombinant FGF8 and FGF15. Non-treated cells were used as a control. The mitotic index was calculated by dividing the number of PH3^+ ^cells with the total number of cells (Hoechst labeled nuclei). Bars in the graph represent the standard deviation. The average number of nuclei and PH3^+ ^cells for each sample/350 μm^2 ^were as follow. T0: 113.5 ± 5 nuclei and 6.25 ± 2.5 PH3^+ ^cells. At 24 hours: control, 226.75 ± 4 nuclei and 20.5 ± 3.8 PH3^+ ^cells; FGF8, 297.5 ± 12.5 nuclei and 48.3 ± 1.5 PH3^+ ^cells; FGF15, 113 ± 10 nuclei and 8.25 ± 2.5 PH3^+ ^cells. At 48 hours: control, 79.6 ± 3 nuclei and 8.25 ± 1 PH3^+ ^cells; FGF8, 164 ± 11 nuclei and 29 ± 3 PH3^+ ^cells; FGF15, 44 ± 3 nuclei and 3.4 ± 0.5 PH3^+ ^cells.

First, we began titrating the FGFs to find concentrations that led to robust changes in the levels of pERK (42/44). We compared 5 ng/ml with 50 ng/ml and found that they led to similar results, with the higher concentration yielding easily detectable levels of pERK (42/44) (Figure 7a, b; Additional file 7c, d). We chose to focus on 50 ng/ml.

We found several qualitative differences in the response to FGF8 and FGF15. First, while both ligands induced a rapid (5 minutes) increase in pERK, their levels decreased more rapidly in the FGF15-treated samples (Figure 7a, b). We used recombinant FGF2 as a control, because it is a known mitogen for cortical progenitors [60,61], and as an activator of the ERK kinase pathway (Additional file 7). FGF2 showed similar results as FGF8 (Figure 7; Additional file 7).

While both FGF8 and FGF2 induced the phosphorylation of AKT (pAKT; Figure 7b, and Additional file 7a, respectively), FGF15 did not (Figure 7a). Activation of ERK and AKT, through phosphorylation, results in the phosphorylation and subsequent inhibition of GSK3 activity [62,63]. FGF8 and FGF2 increased levels of pGSK3 (Figure 7d, and Additional file 7b, respectively), whereas FGF15 treatment did not increase pGSK3 (Figure 7c). As GSK3 is an inhibitor of the Wnt/β-catenin pathway [64], these results suggest that FGF2/8 may repress Wnt signaling more than FGF15 through this mechanism.

FGFs promote protein translation and cell growth through the mTor/S6 pathway [65,66]. FGF8- and FGF2-treated cells increased phosphorylation of S6 protein, albeit with slower kinetics than pERK and pAKT (Figure 7d, and Additional file 7b, respectively). On the contrary, FGF15 did not show this increase; in fact, we observed a reduction of S6 phosphorylation in the first 15 minutes (Figure 7c).

Finally, we compared the effects of FGF15 and FGF8 recombinant proteins on proliferation and differentiation of cortical progenitors *in vitro*. We treated primary E12.5 cortical cultures grown *in vitro *for 24 and 48 hours with either FGF15 or FGF8. While FGF8 induced an approximately 1.7-fold increase in the mitotic index (assessed by comparing the ratio of PH3^+^/total cells in FGF8-treated and untreated cortical cultures), FGF15 caused an approximately 0.25-fold reduction in the mitotic index (Figure 7e–j, and quantification in 7k). This result is consistent with the increased proliferation of the cortical progenitors we observed in the *Fgf15 *mutants, and may reflect the distinct effects of FGF15 on the phosphorylation of ERK (42/44), AKT, GSK3 and S6. We did not test these cultures for apoptosis; however, we have not observed a change in the number of apoptotic cells in the *Fgf15 *mutants (data not shown). A more detailed analysis is required to determine the role of FGF15 on neural progenitor apoptosis.

## Discussion

Here we present the first evidence that *Fgf15*(*19*), another *Fgf *expressed in the rostral patterning center, has telencephalic functions that oppose *Fgf8 *and *Fgf17*. These dichotomous phenotypes include effects on proliferation and on the expression of genes regulating the rostro-caudal patterning of the telencephalon. In addition, FGF15 promotes the neuronal differentiation of cortical progenitor cells. Therefore, we propose that FGF15 is a secreted negative modulator of FGF8/17 signaling outputs. Below, we discuss the ramifications of this property and insights into the potential mechanisms by which FGF15 regulates the development of the neocortex and may modulate FGF8/17 functions.

### Roles of FGF15, FGF8 and SHH in establishing and maintaining the rostral patterning center

**Figure 8:**
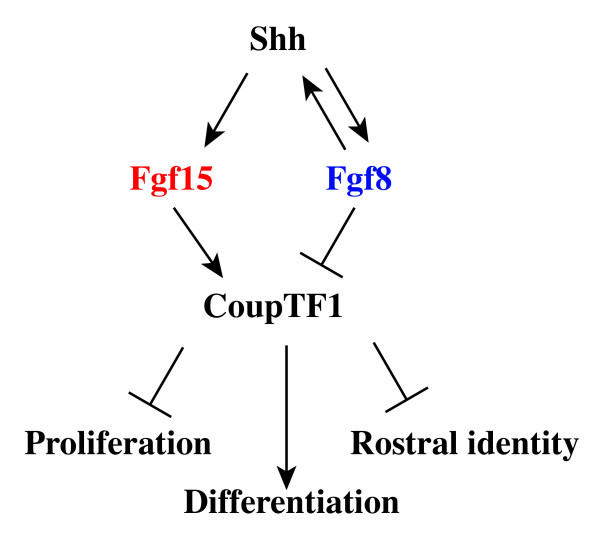
**Model of genetic interactions upstream and downstream of *Fgf15 *and *Fgf8 *within embryonic telencephalon. ***Shh *promotes *Fgf15 *expression (Addition file 2) [6,20,35], and maintains *Fgf8 *expression [68]. *Fgf8 *is required for *Shh *induction [22]. *Fgf15 *activates, whereas *Fgf8 *represses, expression of *CoupTF1 *(among other genes), which represses proliferation and promotes differentiation and caudal fate [37,38].

The vertebrate rostral patterning center expresses multiple *Fgf *genes: *3*, *8*, *15*, *17 *and *18 *[9-14,16,19,67]. It is unknown what induces expression of the earliest *Fgf *(*Fgf8*) in the anterior neural ridge. However, *Fgf8 *is positively upstream of *Fgf17 *and *Fgf18 *(Additional file 1q-r') [17], whereas it appears to repress *Fgf15 *in the rostral midline (Figure 1c, d). In the *Fgf8*^*Null*/*Neo*^severe hypomorph, *Fgf15 *expression remains strong, albeit in a reduced area (Figure 1c, d), perhaps secondary to the reduced proliferation in this region [22]. On the other hand, induction/maintenance of *Fgf15 *expression clearly depends on SHH function (Additional file 2) [6,20,35], as does maintenance of *Fgf8 *expression [68]. *Fgf15 *does not appear to have a major role in regulating expression of *Spry2*, an intracellular antagonist of FGF8 signaling (Figures 1g, j and 6a–l). On the other hand, there is maybe a subtle increase in *Fgf8 *expression in the *Fgf15 *mutant (Figure 1g, h); a more quantitative analysis will be needed to verify this observation. Thus, the rostral patterning center has parallel signaling capabilities, one dominated by FGF8 and the other dominated by FGF15, which depends on SHH and not FGF8 (see schema in Figure 8). Currently, functions for FGF3 and FGF18 in the mammalian rostral patterning center are not known, whereas FGF3 and FGF8 have redundant functions in zebrafish [67].

### Patterning the rostroventral telencephalon: FGF15 and FGF8/17 are competing signals

FGF8 and FGF17 promote rostral telencephalic fate, in part by repressing expression of *CoupTF1*, *Fgfr3 *and *Emx2*, and promoting expression of *Er81*, *Erm*, *Pea3*, *Mest *and *Sp8 *[12,17,21,22,24,25,41]. The dorsal patterning center, through its expression of *BMP*s and *Wnt*s [12,69], can, in principle, repress the rostral patterning center. BMPs can repress *Fgf8 *expression [68]; BMP and Wnt positively regulate *Emx2 *expression [70] and reduced *Emx2 *leads to expansion of *Fgf *expression [1,2,17,69]. However, currently, *in vivo *genetic studies have principally linked Wnt signaling to hippocampal specification [1,2] and BMP signaling to choroids plexus specification [71,72], so it remains unclear to what extent the dorsal patterning center participates in neocortical patterning.

Here we show that FGF15 functions, at least in part, to repress rostral telencephalic fate, through promoting *CoupTF1 *and repressing *Mest *and *Sp8 *(Figure 2). Thus, FGF15, in conjunction with the putative role of the dorsal patterning center, participates in repressing the size, and perhaps the nature, of the rostral telencephalon.

FGF8 is also essential for inducing the ventral telencephalon, through promoting expression of *Nkx2.1 *and *Shh *[22,73]. While *Fgf15 *is expressed more ventrally than *Fgf8 *within the rostral patterning center (septum) (this paper) [17], to date we have detected only mild subpallial or septal hypoplasia (Additional files 3 and 4; data not shown). On the other hand, reduced *Fgf19 *expression in zebrafish (homologue of mouse *Fgf15*), results in greatly reduce expression of subcortical molecular markers [34], perhaps suggesting divergent functions for these genes in fish and mammals. In the mouse *Fgf15 *mutant, there are reduced numbers of cortical GABAergic interneurons in the dorsomedial cortex (Additional file 4); at this point we can not distinguish whether this is a defect secondary to the abnormal cortical environment, or secondary to abnormal subpallial development (cortical interneurons are generated in the basal ganglia anlage).

### Where and when does FGF15 affect telencephalic development?

We propose that *Fgf15 *expression in the rostral patterning center has a profound role in telencephalic patterning during early neurulation based on the reduction of *CoupTF1 *expression at E9.5 (Figure 1e, f). At this stage, the distribution of *Fgf15 *mRNA is in a rostrocaudal gradient whose pattern is roughly complementary to the expression of *CoupTF1 *mRNA (Figure 1a, c; Additional file 1a–d). Assuming that the RNA and protein distributions are similar at E9.5, then FGF15 secretion could promote *CoupTF1 *expression in the most rostral domain. At later stages, *Fgf15 *expression in the septum and *CoupTF1 *expression in the cortex are far apart; therefore, it seems unlikely that loss of *Fgf15 *from the septum at these stages contributes to *CoupTF1 *reduction, especially in the dorsolateral cortex. However, *Fgf15 *is also expressed at the PSB, and in the CGE (Additional file 1g–j); *Fgf15 *from those sites could contribute to maintaining *CoupTF1 *cortical expression at later developmental stages. This suggests that FGF15 could play a key role in the putative patterning center at the PSB. This is the first evidence for a role of a secreted factor expressed in this putative patterning center [7,8]. *Fgf15 *is also expressed in the lateral and medial ganglionic eminences (LGE/MGE) and the thalamus; currently, we have not detected phenotypes in these structures.

### Establishing the balance between proliferation and differentiation: distinct roles of FGF15(19) in fish and mammals

The most overt phenotype in the *Fgf15 *mutants was reduced neurogenesis and increased proliferation in the cortex (Figures 3 and 4). Consistent with this, *in vitro *cultures of cortical progenitors with recombinant FGF15 have reduced neuronal proliferation (Figure 7). These data differ from the results obtained by reducing expression of the zebrafish *Fgf15 *homologue (*Fgf19*) with antisense oligonucleotides (morpholinos) [34]. Whereas reduced *Fgf19 *expression led to a decrease in the number of proliferating cells in the embryonic fish brain, *Fgf15*^-/- ^mice showed increased proliferation. These divergent phenotypes could be explained by the different methodologies to reduce gene expression (constitutive deletion mutation versus morpholino), different compensatory responses, or distinct functions of mouse *Fgf15 *and zebrafish *Fgf19*. It is conceivable that the *Fgf15 *deletion produces an amino-terminal fragment with altered signaling properties; however, the deletion does remove exon 3, which encodes the residues predicted to bind heparin sulfate proteoglycans and FGF receptors [32]. Future studies with a *Fgf19 *zebrafish mutant will help resolve whether zebrafish *Fgf19 *and mouse *Fgf15 *indeed have divergent functions that may contribute to the divergent morphogenesis of the fish and mammalian telencephalons.

### Opposite roles of FGF15 and FGF8 in regulating the balance between proliferation and differentiation through controlling *CoupTF1 *levels

We suggest that an important aspect of Fgf15's functions is to promote expression of the *CoupTF1 *transcription factor, because the cortical phenotypes observed through changing *Fgf15*'s expression levels mirror those observed when altering *CoupTF1 *dosage. Increased *CoupTF1 *represses proliferation and promotes neurogenesis, whereas loss of *CoupTF1 *promotes proliferation and represses neurogenesis [38]. As loss of *Fgf15 *expression results in decreased *CoupTF1 *expression as early as E9.5 (Figure 1), we suggest that the *Fgf15 *mutant phenotype is caused, in part, by reduced *CoupTF1 *levels. However, because the neurogenesis phenotype in *Fgf15 *mutants is more severe than that of the *CoupTF1 *mutants, other factors must contribute.

Unlike FGF15, FGF8 represses *CoupTF1 *[21,22]. Furthermore, FGF8 promotes proliferation in the developing telencephalon [22] (Figure 7), as does FGF2 [14,60,74,75]. Thus, while the *in vivo *functions of certain FGFs (FGF2 and FGF8) promote proliferation in the developing telencephalon, FGF15 has the opposite role. Therefore, FGF15 and FGF8 provide opposing signals from the rostral patterning center that control the balance of proliferation and differentiation. One can imagine how differential modulation of these signals during development, disease, or evolution will have profound effects on cortical size, thickness and regional fate.

### Interplay between FGF15 and FGF8/17 signaling: complementary effects on *Spry *and *Fgf receptor *expression

FGF15 and FGF8/17 are believed to signal through several receptor tyrosine kinases (FGFR1-4) that are negatively modulated by several mechanisms. *Spry1-4 *encode FGF-induced cytoplasmic repressor of FGF-signaling [53,76]. While *Spry *expression is clearly reduced in the *Fgf8 *mutant forebrain [17,22], we did not detect a reduction in *Spry *expression in the rostral patterning center/septum of the *Fgf15 *mutants (Figures 1i, j and 6e–l). Fgfr3 appears to have atypical signaling outputs. In fact, *Fgfr3 *mutant mice have increased proliferation and reduced differentiation of chondrocytes [77,78], and pancreatic cells [79]. *Fgf8 *mutants have increased *Fgfr3 *expression [21], whereas the *Fgf15 *mutants show the opposite phenotype (Figure 6). Thus, we propose that FGF15 is a secreted modulator of several cellular processes that are promoted by FGF8.

### Distinct effects of FGF15 and FGF8 on signalling pathways

Towards elucidating the differential effects of reducing FGF15 and FGF8, we compared the effects of recombinant FGF15/19 and FGF8 on the levels of phosphorylated forms of ERK (42/44), AKT, S6 and GSK3 in primary cultures made from E12.5 mouse cortex (Figure 7). We demonstrated that while FGF15 phosphorylation of ERK kinase (42/44) was transient (approximately 15 minutes), FGF8 phosphorylation of ERK was sustained over the time of the experiment (1 hour). The duration of FGF-signaling is associated with distinct cellular responses [80]. For example, in fibroblasts, sustained activation of ERK correlates with S phase entry [81-84], while in PC12 cells, sustained, but not transient, activation of ERK induces differentiation into sympathetic-like neurons [85-88].

FGF15 and FGF8 also had distinct effects on the levels of pAKT and pS6. While FGF8 increased the levels of both pAKT and pS6 phosphorylation, FGF15 did not increase pAKT, and appeared to reduce pS6. The differences observed in the phosphorylation of the effector kinases of the FGF signaling pathway may contribute to the opposite phenotype of the FGF15 and FGF8 mutants observed *in vivo *and *in vitro*.

The simplest model to account for the signaling differences between FGF8 and FGF15 would be that they differentially activate/repress FGF, or other, receptors. While studies have established the effects of distinct ligands and receptor combinations on mitogenesis and *in vitro *binding [14,55,57,89], definitive elucidation of the *in vivo *biochemistry is lacking, particularly for FGF15. Although FGF15/19 preferentially binds FGFR4 [33,55-57], this receptor does not appear to be expressed in the forebrain until approximately E14.5 (Additional file 6). In addition, FGF15/19 binds Klotho-β, which acts as a co-receptor. We have not observed any expression of Klotho-β in the forebrain at E12.5 and E14.5 (data not shown). Therefore, it is likely that FGF15 functions by regulating other FGF receptors. Therefore, additional analysis is needed to establish how FGF8 and FGF15 signal to elucidate how they differentially regulate the balance between proliferation and differentiation.

## Conclusion

We provide novel evidence that FGF15 and FGF8 have opposite functions in mouse forebrain development. In the cortex, FGF15 suppresses proliferation and promotes differentiation, expression of *CoupTF1 *and caudoventral fate. Furthermore, using primary cultures and recombinant proteins, we demonstrate that FGF15 and FGF8 differentially phosphorylate ERK (p42/44), AKT and S6. Finally, we show that FGF15 blocks neural proliferation in these cortical cultures.

## Materials and methods

### Mice

The *Fgf15*^-/- ^[32], *Fgf8*^*Null*/+ ^and *Fgf8*^*Neo*/+ ^[22], *Shh*^± ^[90], *BAT-gal *[52] and *RARE-LacZ *[47] strains were maintained on a mixed C57BL6/CD1 genetic background. Screening of the mutant alleles was performed by PCR genotyping as described previously [22,32,90]. Noon on the day of the vaginal plug was considered as E0.5. Mouse colonies were maintained at the University of California, San Francisco, in accordance with National Institutes of Health and UCSF guidelines.

### Histology

Pregnant females were deeply anesthetized with CO_2 _and sacrificed by cervical dislocation. The embryos were dissected and the brains were fixed by immersion in 4% paraformaldehyde in phosphate buffered saline. The tissue was cryoprotected by immersion in 30% sucrose/phosphate buffered saline, embedded in OCT (Tissue-Tek, Sakura Finetek, Torrance, CA, USA), and cryostat sectioned (10–20 μm).

Immunofluorescence on cryostat sections was performed as previously described [22]. The antibodies used were as follows: mouse anti-Tuj1 (1:1,000; Covance, Princeton, NJ, USA), rabbit anti-PH3 (1:200; Upstate/Millipore, Billerica, MA, USA), mouse anti-BrdU (1:100; Becton Dickinson, Franklin Lakes, NJ, USA), rat anti-BrdU (1:100; Abcam, Cambridge, MA, USA). Goat anti-rabbit, goat anti-mouse and goat anti-rat secondary antibodies, conjugated with either Alexa 488 or Alexa 594 (1:300, Molecular Probes/Invitrogen, Carlsbad, CA, USA), were used at a dilution of 1:300.

*In situ *RNA hybridization on cryostat sections was performed as previously described [22]. Comparison of gene expression changes between brains of different genotypes was performed by matching the planes of section to the best of our abilities, using multiple anatomical features. Whenever possible, this was performed for multiple planes of section for each gene, and from at least two brains for each genotype.

### Cell cycle analysis

For the cell cycle kinetic analysis, a single injection of IdU (50 μg/g; Sigma, St Louis, MO, USA) was administered to pregnant females, carrying either E12.5 or E14.5 embryos, at T = 0. This was followed at T = 1.5 hours by a single injection of BrdU (50 μg/g; Sigma). Mice were sacrificed at T = 2 hours.

For the quantification of the numbers of cells exiting the cell cycle (Q fraction), IdU was administered as a single injection at T = 0. This was followed at T = 1.5 hours by sequential injections of BrdU every 3 hours, for a total of 15 hours. Mice were sacrificed at 0.5 hours after the last injection.

For the calculation of BrdU/IdU labeling index, we sampled two 100 μm bins spaced 200 μm apart in the ventro-lateral region of the cortex (rectangles in Figure 4 and Additional file 5). Images were acquired using a confocal microscope (Radiance 2000, Bio-Rad, Hercules, CA, USA) with a 20× or a 40× objective. BrdU/IdU positive cells (red channel) and BrdU positive cells (red channel) were counted together with the total number of cells for each bin (calculated by the number of Hoechst labeled nuclei). The experimenter was blind during sampling, image analysis, data collection and statistical analysis. Digitized images were imported into Phostoshop CS3 (Adobe) for counting. Statistical analysis was performed with SPSS (SPSS) and data plotted using Excel (Microsoft) and the significance level was taken as *p *< 0.05.

A total of three cases were counted in sections spaced evenly through the rostrocaudal cortex. The cell cycle length was calculated using the paradigm described in [91].

### FGF15 adenovirus preparation and FGF15 protein purification

The adenovirus containing the *Fgf15 *coding region with a (His)_6 _tag was the gift of Dr Steven Kliewer (UT Southwestern Medical Center, Dallas, TX, USA). The FGF15 adenovirus was grown in HEK293 cells (ATCC: CRL 1573) and titrated onto the same cells. 1.2 × 10^7 ^COS7 cells (ATCC: CRL 1673) were plated in T150 flasks (6 flasks for each protein preparation). The cells were infected with adenovirus at a multiplicity of infection of 2 for 2 hours in Dulbecco's modified Eagle's medium (DMEM)/2% fetal calf serum. After the infection, the medium was changed with DMEM without fetal calf serum. Three days post-infection the supernatant was collected, equilibrated with a 10× solution of 500 mM Tris/HCl pH8, 1 M NaCl, 100 mM Imidazole, centrifuged 20 minutes at 10,000 g, and passed through a 0.8 ml nickel agarose column (Ni-NTA Agarose, Quiagen, Valencia, CA, USA). The column was washed with 25 column volumes of 50 mM Tris/HCl pH 8, 0.25 M NaCl, 0.1% NP-40, 10 mM Imidazole, and then with 25 column volumes of 20 mM Tris/HCl pH 8, 0.25 M NaCl, 10 mM Imidazole. The His-tagged FGF15 protein was eluted in 20 mM Tris/HCl pH 8, 0.25 M NaCl, 0.5 M Imidazole. The eluate was passed through a PD MidiTrap G-25 desalting column (GE Healthcare, Piscataway, NJ, USA) following the manufacturer's protocol, eluted in 10 mM Tris/HCl pH 7.5, 135 mM NaCl, 5 mM KCl, 1 mM MgCl_2_, and finally concentrated with the Amicon Ultra-4 3 k centrifugal filter device (Millipore, Billerica, MA, USA). The purity of the protein preparation was assessed by Coomassie Blue staining and by immunoblotting (western blot) using an anti-FGF15 polyclonal antibody (SC 16816, Santa Cruz Biotechnology, Santa Cruz, CA, USA).

### Primary cell culture and treatment with recombinant FGFs

Wild-type CD1 brains (E12.5) were used to prepare primary cortical cultures. The brains were removed in ice-cold Hank's solution (HBSS; Invitrogen, Carlsbad, CA, USA), and the cortices dissected, after removing the external membranes. The samples were mechanically dissociated in DMEM/10% fetal bovine serum (FBS; Invitrogen) and the single cell suspensions were plated at a density of 2.5 × 10^5 ^cells per cm^2 ^in two-well chamber slide (Lab-Tek, Nalgene, Rochester, NY, USA). We were careful to plate the cells at the same density in all of the wells. After 16 hours the medium was replaced with DMEM/5% FBS and finally changed with DMEM/0.5% FBS after 24 hours. The cells were starved in DMEM/0.5% FBS for 24 hours before the treatment with the recombinant proteins. The mouse FGF15 recombinant protein was added to the cells at a concentration of 50 ng/ml in DMEM/0.5% FBS/5 μg/ml Heparin. This protein concentration corresponds to 1.7 pmol/ml for FGF2 and 2 pmol/ml for FGF15 and FGF8. The same protocol was used for human FGF2, FGF8 and FGF19 recombinant proteins (Fitzgerald Industries International, RDI, Concord, MA, USA).

For counting the cultured cells, we used an unbiased method that gave every cell an equal chance of being sampled. We defined a systematic series of fields of view in the culture area to cover the surface of the well. The experimenter was blind to sample identity during sampling, image analysis, data collection and statistical analysis. Images were acquired on a fluorescence microscope with a 20× objective. We sampled at least two different wells for each time point obtained from four independent experiments. Digitized images were imported into Phostoshop CS3 (Adobe) for counting. Statistical analysis was performed with SPSS (SPSS) and data plotted using Excel (Microsoft) and the significance level was taken as *p *< 0.05.

### Immunoblotting

Cells from primary E12.5 cortical cultures were lysed in radioimmunoprecipitation buffer (RIPA: 50 mM Tris/HCl pH 7.5, 150 mM NaCl, 0.5% sodium deoxycolate, 0.1% SDS, 1% NP-40) supplemented with protease inhibitors (Protease Inhibitor Cocktail, Roche, Indianapolis, IN, USA) and phosphatase inhibitors (Phosphatase Inhibitor Mix 1 and 2, Sigma). Protein samples (10 μg each) were resolved on polyacrylamide gels and transferred to nitrocellulose membranes by electroblotting. Membranes were pre-incubated in 5% nonfat dry milk in Tris buffered solution (TBS)/0.1% Tween-20 (TBST) and then incubated O/N with the primary antibodies in TBST/5% bovine serum albumin in TBST. The primary antibodies were: rabbit-anti phospho-p44/42 (Thr202/Tyr204) MAPK (1:1,000; Cell Signaling, Boston, MA, USA), rabbit anti-phospho-Akt (Ser 473) (1:500; Cell Signaling), rabbit anti-phospho-GSK3α/β (Ser 21/9) (1:1,000; Cell Signaling), rabbit anti-phospho-S6 ribosomal protein (Ser235/236) (1:1,000; Cell Signaling), and mouse anti-α-tubulin (1:10,000; Sigma). Membranes were probed with the appropriate goat horseradish peroxidase-conjugated antibodies (1:500; Biorad, Hercules, CA, USA) and developed using ECL reagents (GE Healthcare). Each experiment was repeated at least three times. Quantification of optical density was done using NIH ImageJ software; the intensity of the bands was normalized with respect to the intensity of the α-Tubulin band. The numbers indicated in Figure 7 and Additional file 7 represent the fold-induction, or reduction, from the time point before FGF addition (T0).

## Abbreviations

BMP: bone morphogenetic protein; BrdU: bromo-deoxyuridine; CGE: caudal ganglionic eminence; DMEM: Dulbecco's modified Eagle's medium; E: embryonic day; FBS: fetal bovine serum; FGF: fibroblast growth factor; FGFR: FGF receptor; IdU: iodo-deoxyuridine; MAPK: mitogen activated protein kinase; PH3: phospho-histone H3; PSB: pallium-subpallium boundary; SHH: sonic hedgehog; SVZ: subventricular zone; VZ: ventricular zone.

## Competing interests

The authors declare that they have no competing interests.

## Authors' contributions

UB conceived of the studies, designed and carried out the experiments, analyzed and interpreted the data, and drafted the paper. IC helped with the *in vitro *cultures and data analysis. JL helped with the *in situ *RNA hybridization. CM provided the *Fgf15 *mouse strain. JLRR conceived of the studies, coordinated them, analyzed and interpreted the data, and drafted the paper. All authors read and approved the final manuscript.

## Supplementary Material

Additional file 1 *Fgf15 *and *Fgf17 *expression in *Fgf8*^*Null*/*Neo *^mutants and *Fgf17 *expression in *Fgf15*^-/- ^mutants. (a-p') *In situ *hybridization of *Fgf15 *in *Fgf8*^*Null*/*Neo *^embryos. E9.5: wild type (a-d); mutant (a'-d'); (a"-d", a"'-d"') show higher magnification images; horizontal sections. E12.5: wild type (e-j); mutant (e'-j'); coronal sections. E14.5: wild type (k-p); mutant (k'-p'); coronal sections. (q-r') *In situ *hybridization of *Fgf17 *in *Fgf8*^*Null*/*Neo *^embryos at E12.5: wild type (q, r); mutant (q', r'); coronal sections. (s-t') *In situ *hybridization of *Fgf17 *in *Fgf15*^-/-^embryos. E14.5: wild type (s, t); mutant (s', t'); coronal sections. Bar in (a, e, k) is 200 μm. Click here for file

Additional file 2 *Fgf15 *and the SHH pathway. (a-p') Analysis of *Fgf15 *expression in the *Shh *mutant. E12.5: *Shh*^± ^(a-o); *Shh*^-/- ^mutant (a'-o'); coronal sections. E14.5: *Shh*^± ^(b-p); *Shh*^-/- ^mutant (b'-p'); coronal sections. (q-r') Expression of SHH downstream effector *Gli3 *in the *Fgf15 *mutants in coronal sections at E12.5 (q wild type, q' *Fgf15*^-/-^mutant) and E14.5 (r wild type, r' *Fgf15*^-/- ^mutant). Bar in (a) and (b) is 200 μm. Click here for file

Additional file 3 *Fgf15 *mutant embryos morphology. The morphology of *Fgf15*^-/- ^mutant embryos was compared to the wild type. (a, b) Examples of lateral views of *Fgf15 *mutant and wild-type embryos at E10.5. **-**(c, d) Comparison of dorsal views of *Fgf15 *mutant and wild-type dissected brains at E14.5. (e, f) β-III-Tubulin immunostaining on coronal hemisections of E14.5 brains. Click here for file

Additional file 4 Analysis of markers of the basal ganglia and basal ganglia-derived cortical interneurons in the *Fgf15 *mutants. (a-j) *In situ *hybridization on coronal sections at E14.5: *Gad67 *(a-d), *Reelin *(e, f), *Nkx2.1 *(g, h), *Pax6 *(i, j). Ncx, neocortex; Pcx, piriform cortex; Se, septum; St, striatum. Arrows in (a, b) indicate reduced cortical interneurons in the dorsomedial cortex; arrowheads in (a, b) indicate suggestive evidence for increased *Gad67 *expression in the deep tangential migration. Arrows in (c, d) show reduced thickness in the mantle zone of the ventral septum and piriform cortex. Bar in (a, c) is 200 μm. Click here for file

Additional file 5 Analysis of proliferation and cell cycle in the *Fgf15 *mutants. (a-c) The number of progenitors undergoing mitosis at E12.5 was determined by PH3 immunofluorescence (n = 3; *p *= 0.0024, Student's *t*-test). (d-f) the analysis of the cell cycle length was performed at E12.5 by double labeling with IdU and BrdU. Click here for file

Additional file 6 Analysis of FGF signaling components in the *Fgf15 *mutants. *In situ *hybridization on coronal sections. (a-d') *Fgfr1 *at E12.5 (a, b), and at E14.5 (c, d); (c', d') show higher magnification images. (e-h') *Fgfr2 *at E12.5 (e, f), and at E14.5 (g, h); (g', h') show higher magnification images. (i-l) *Fgfr3 *at E12.5 (i, j), and at E14.5 (k, l)). (m-n) *Fgfr4 *at E14.5. CP: cortical plate. Bar in (a) is 200 μm. Click here for file

Additional file 7 Analysis of the downstream cytoplasmic effectors induced by FGF2. (a, b): comparison of phosphorylation of four proteins that are modified in response to FGF2. E12.5 primary cortical cultures were starved for 24 hours and treated with recombinant FGF2. Cell lysates were analyzed after 0, 5, 15, 30 and 60 minutes by immunoblotting to detect phosphorylated forms of pERK 42/44 (a, top panel), pAKT (a, middle panel), pS6 (b, top panel), and pGSK3 (b, middle panel). The α-Tubulin antibody (a, b, bottom panels) was used for normalization. Analysis of the phosphorylation of pERK (42/44) induced by FGF8 and FGF15 at 5 ng/ml (c and d, respectively, top panels). The α-Tubulin antibody was used for normalization (c, d, bottom panels). The numbers under the bands indicate the fold-induction, or reduction, with respect to T0. Click here for file
