# Correction: FGF15 promotes neurnogenesis and opposes FGF8 function during neocortical development

**DOI:** 10.1186/1749-8104-3-31

**Published:** 2008-11-05

**Authors:** Ugo Borello, Inma Cobos, Jason E Long, John R McWhirter, Cornelis Murre, John LR Rubenstein

**Affiliations:** 1 Nina Ireland Laboratory of Developmental Neurobiology, Department of Psychiatry, University of California, San Francisco, CA 94143, USA; 2 Department of Biology, University of California, San Diego, CA 92093, USA; 3 ICREA and Department of Cell Biology, University of Barcelona. Avda. Diagonal, 08028 Barcelona, Spain; 4 Genentech, Inc., DNA Way, South San Francisco, CA 94080, USA; 5 Regeneron Pharmaceuticals, 777 Old Saw Mill River Road, Tarrytown, New York 10591, USA

## Correction

After publication of this work [1], we noted that we inadvertently failed to include the complete list of all co-authors. The full list of authors has now been added and the Authors' contributions and Competing interests section modified accordingly.

## Competing interests

The authors declare that they have no competing interests.

## Authors' contributions

UB co-conceived of the studies, designed and carried out the experiments, analyzed and interpreted the data, and drafted the paper. IC helped with the *in vitro *cultures and data analysis. JL helped with the *in situ *RNA hybridization. CM and JRM provided the *Fgf15 *mouse strain. JLRR co-conceived of the studies, coordinated them, analyzed and interpreted the data, and drafted the paper. All authors read and approved the final manuscript.
