# Supplementary figures and images for: FGF15 promotes neurogenesis and opposes FGF8 function during neocortical development
Source: Neural Dev. 2008 Jul 14;3:17. doi: 10.1186/1749-8104-3-17 (PMC2492847; doi:10.1186/1749-8104-3-17)

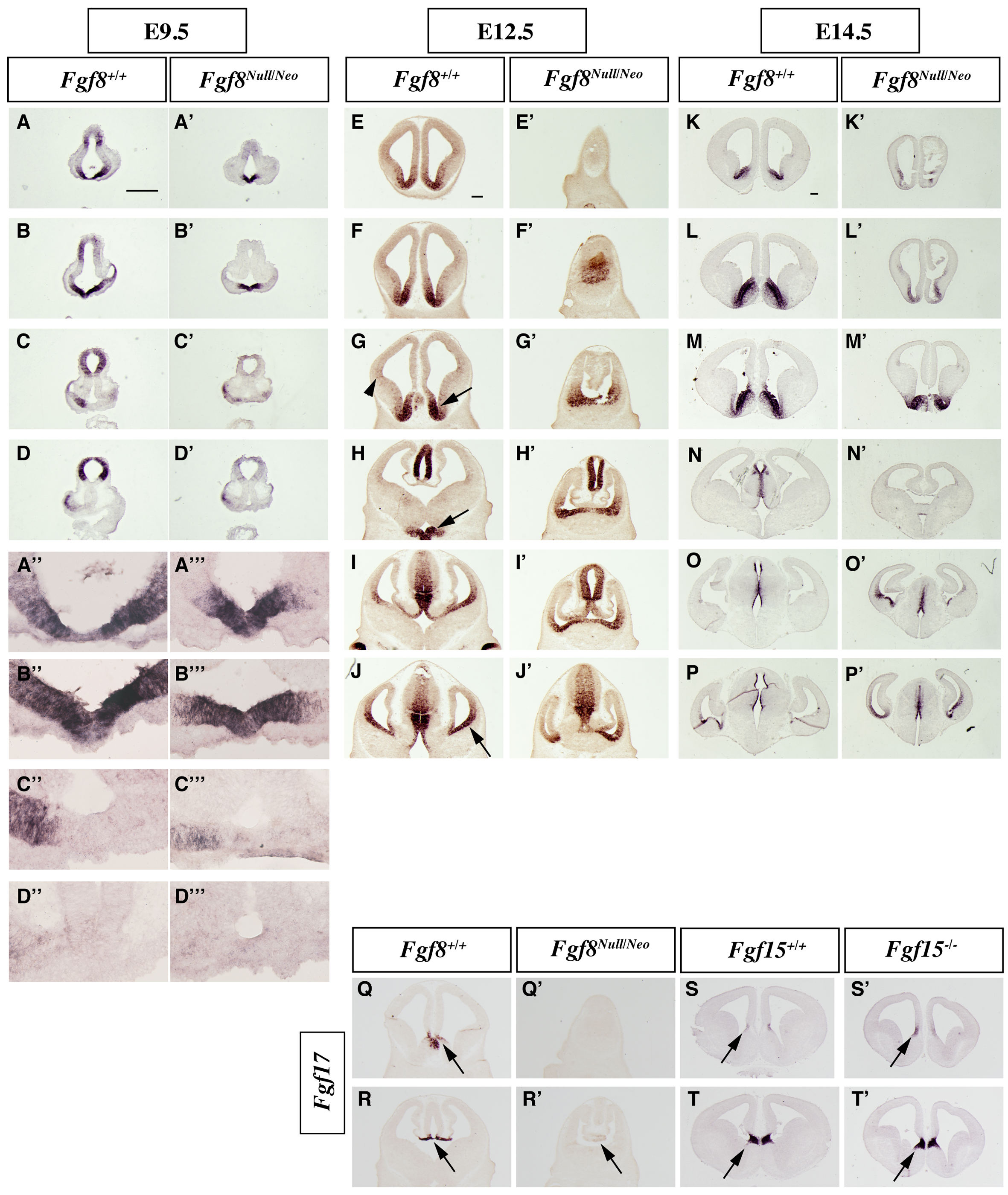

Supplement: Additional file 1 — Fgf15 and Fgf17 expression in Fgf8Null/Neo mutants and Fgf17 expression in Fgf15-/- mutants. (a-p') In situ hybridization of Fgf15 in Fgf8Null/Neo embryos. E9.5: wild type (a-d); mutant (a'-d'); (a"-d", a"'-d"') show higher magnification images; horizontal sections. E12.5: wild type (e-j); mutant (e'-j'); coronal sections. E14.5: wild type (k-p); mutant (k'-p'); coronal sections. (q-r') In situ hybridization of Fgf17 in Fgf8Null/Neo embryos at E12.5: wild type (q, r); mutant (q', r'); coronal sections. (s-t') In situ hybridization of Fgf17 in Fgf15-/-embryos. E14.5: wild type (s, t); mutant (s', t'); coronal sections. Bar in (a, e, k) is 200 μm. [file 1749-8104-3-17-S1.tiff]

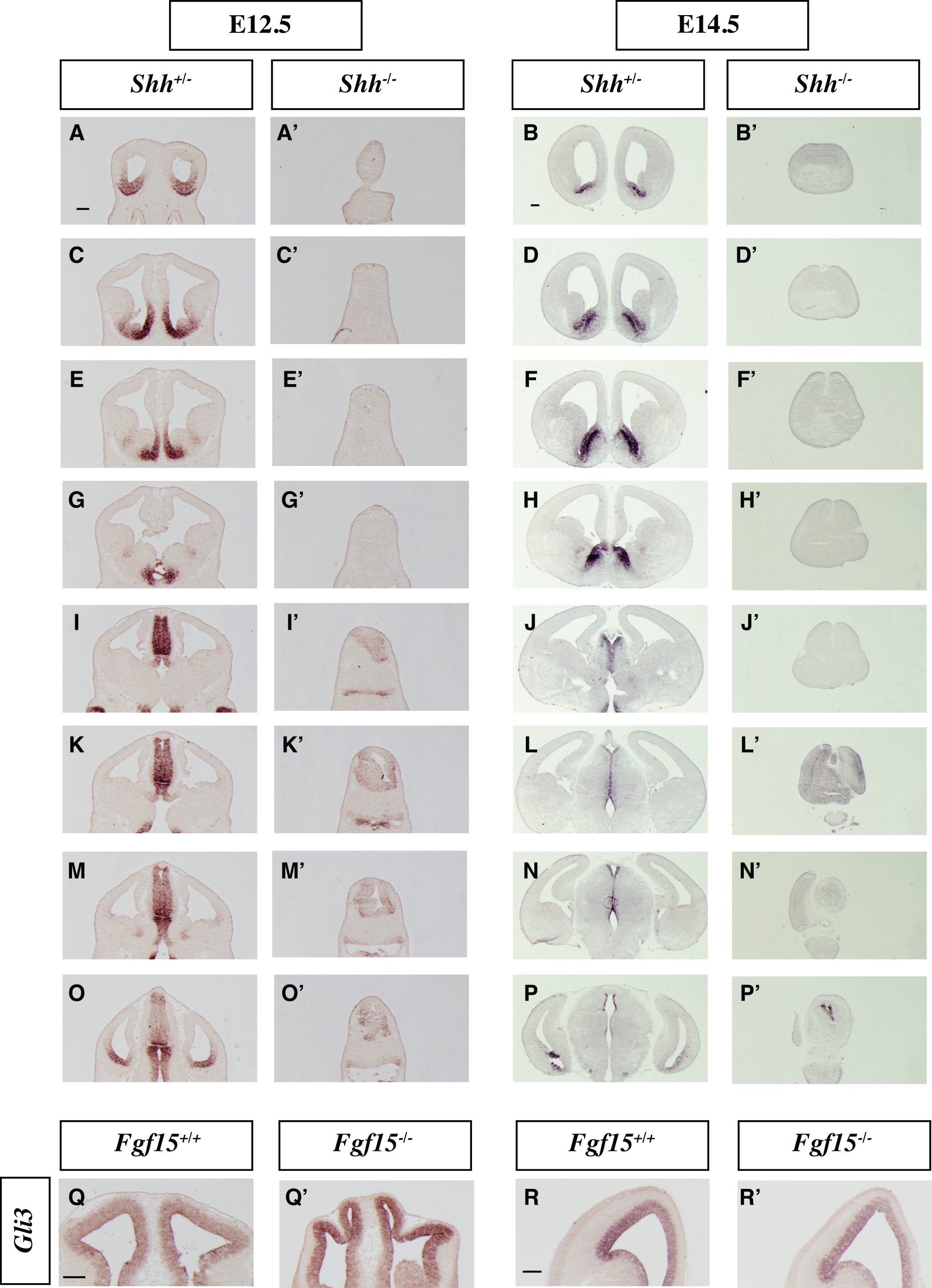

Supplement: Additional file 2 — Fgf15 and the SHH pathway. (a-p') Analysis of Fgf15 expression in the Shh mutant. E12.5: Shh± (a-o); Shh-/- mutant (a'-o'); coronal sections. E14.5: Shh± (b-p); Shh-/- mutant (b'-p'); coronal sections. (q-r') Expression of SHH downstream effector Gli3 in the Fgf15 mutants in coronal sections at E12.5 (q wild type, q' Fgf15-/-mutant) and E14.5 (r wild type, r' Fgf15-/- mutant). Bar in (a) and (b) is 200 μm. [file 1749-8104-3-17-S2.tiff]

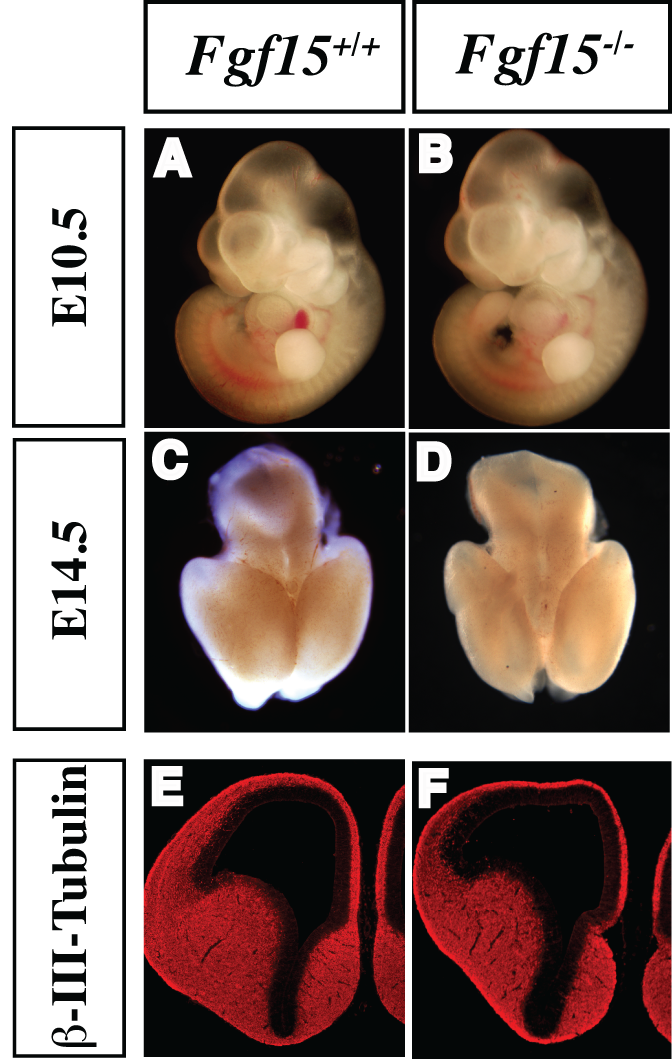

Supplement: Additional file 3 — Fgf15 mutant embryos morphology. The morphology of Fgf15-/- mutant embryos was compared to the wild type. (a, b) Examples of lateral views of Fgf15 mutant and wild-type embryos at E10.5. -(c, d) Comparison of dorsal views of Fgf15 mutant and wild-type dissected brains at E14.5. (e, f) β-III-Tubulin immunostaining on coronal hemisections of E14.5 brains. [file 1749-8104-3-17-S3.tiff]

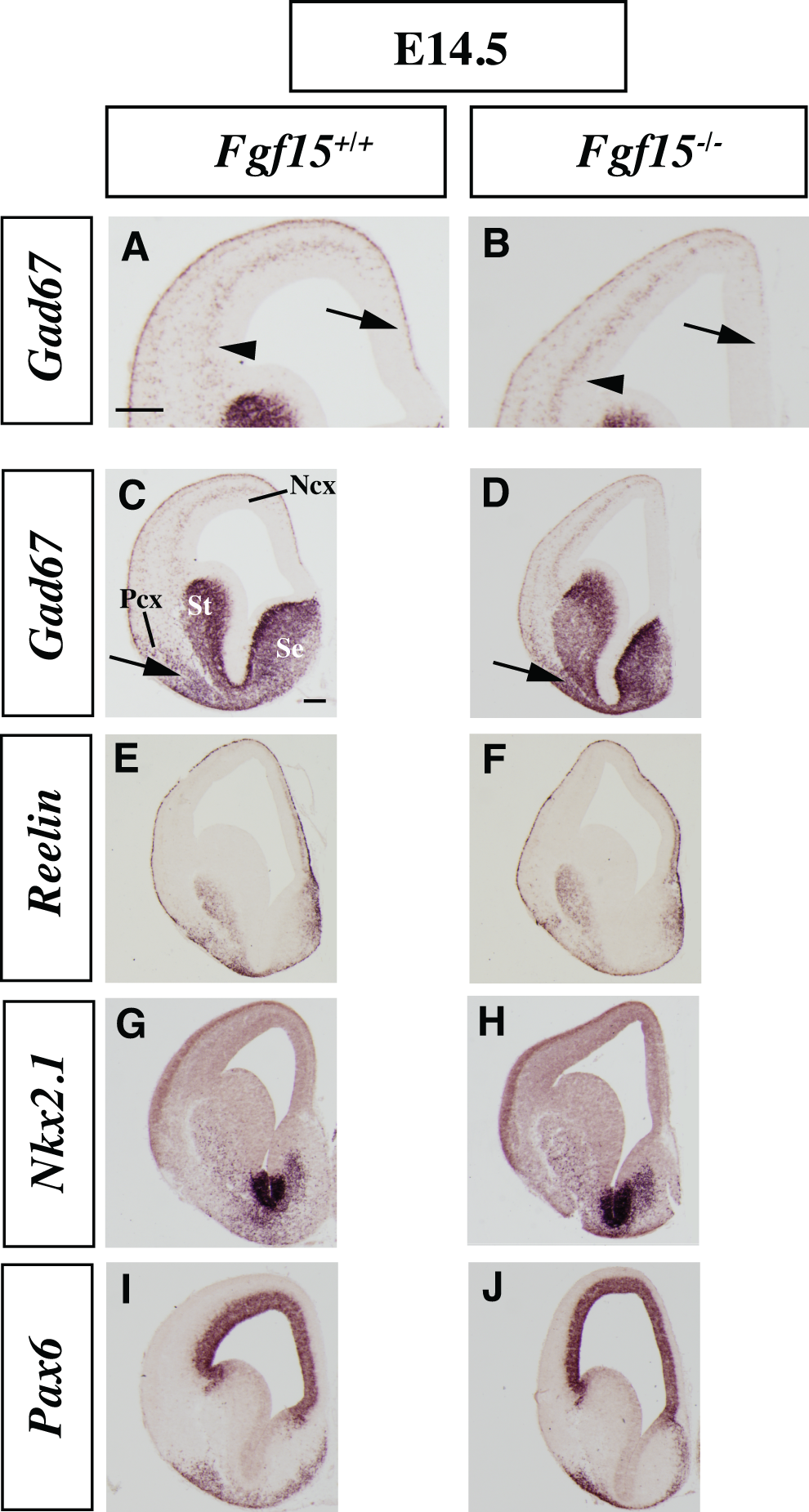

Supplement: Additional file 4 — Analysis of markers of the basal ganglia and basal ganglia-derived cortical interneurons in the Fgf15 mutants. (a-j) In situ hybridization on coronal sections at E14.5: Gad67 (a-d), Reelin (e, f), Nkx2.1 (g, h), Pax6 (i, j). Ncx, neocortex; Pcx, piriform cortex; Se, septum; St, striatum. Arrows in (a, b) indicate reduced cortical interneurons in the dorsomedial cortex; arrowheads in (a, b) indicate suggestive evidence for increased Gad67 expression in the deep tangential migration. Arrows in (c, d) show reduced thickness in the mantle zone of the ventral septum and piriform cortex. Bar in (a, c) is 200 μm. [file 1749-8104-3-17-S4.tiff]

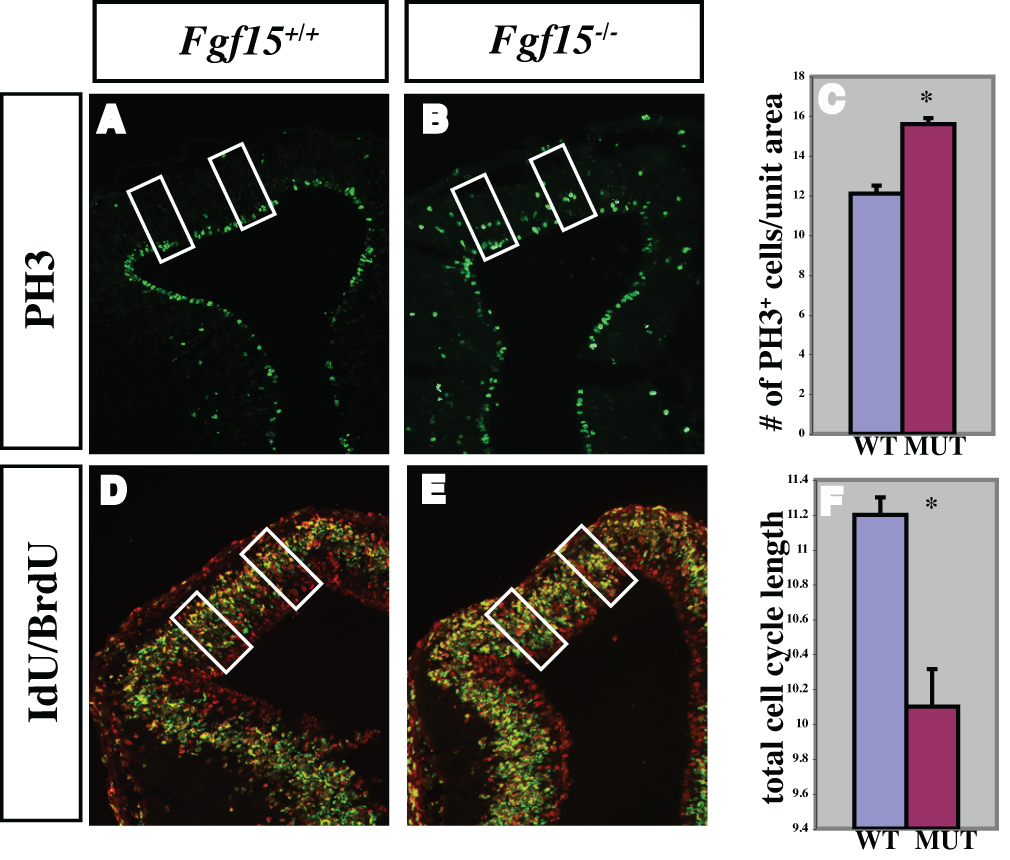

Supplement: Additional file 5 — Analysis of proliferation and cell cycle in the Fgf15 mutants. (a-c) The number of progenitors undergoing mitosis at E12.5 was determined by PH3 immunofluorescence (n = 3; p = 0.0024, Student's t-test). (d-f) the analysis of the cell cycle length was performed at E12.5 by double labeling with IdU and BrdU. [file 1749-8104-3-17-S5.tiff]

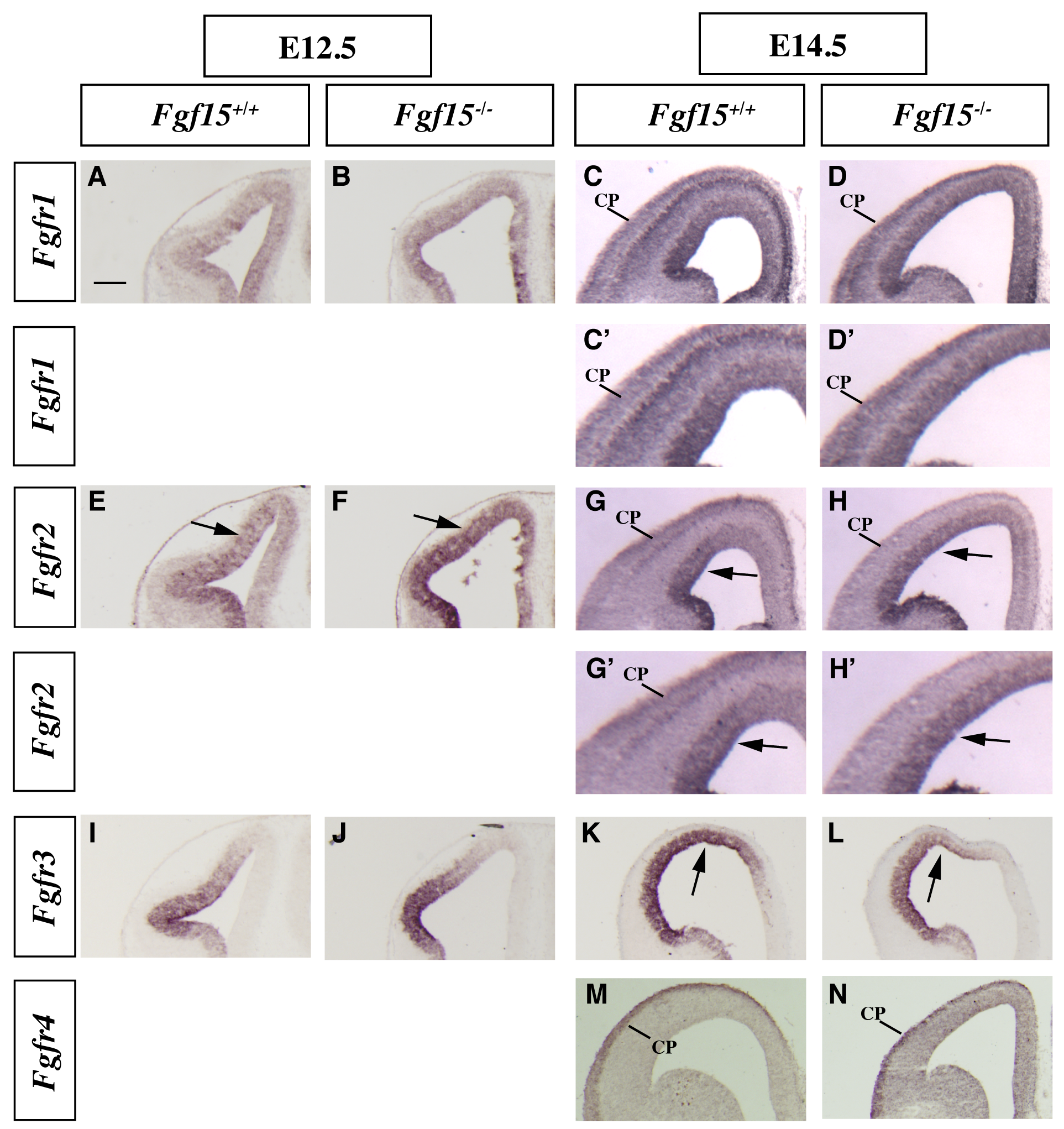

Supplement: Additional file 6 — Analysis of FGF signaling components in the Fgf15 mutants. In situ hybridization on coronal sections. (a-d') Fgfr1 at E12.5 (a, b), and at E14.5 (c, d); (c', d') show higher magnification images. (e-h') Fgfr2 at E12.5 (e, f), and at E14.5 (g, h); (g', h') show higher magnification images. (i-l) Fgfr3 at E12.5 (i, j), and at E14.5 (k, l)). (m-n) Fgfr4 at E14.5. CP: cortical plate. Bar in (a) is 200 μm. [file 1749-8104-3-17-S6.tiff]

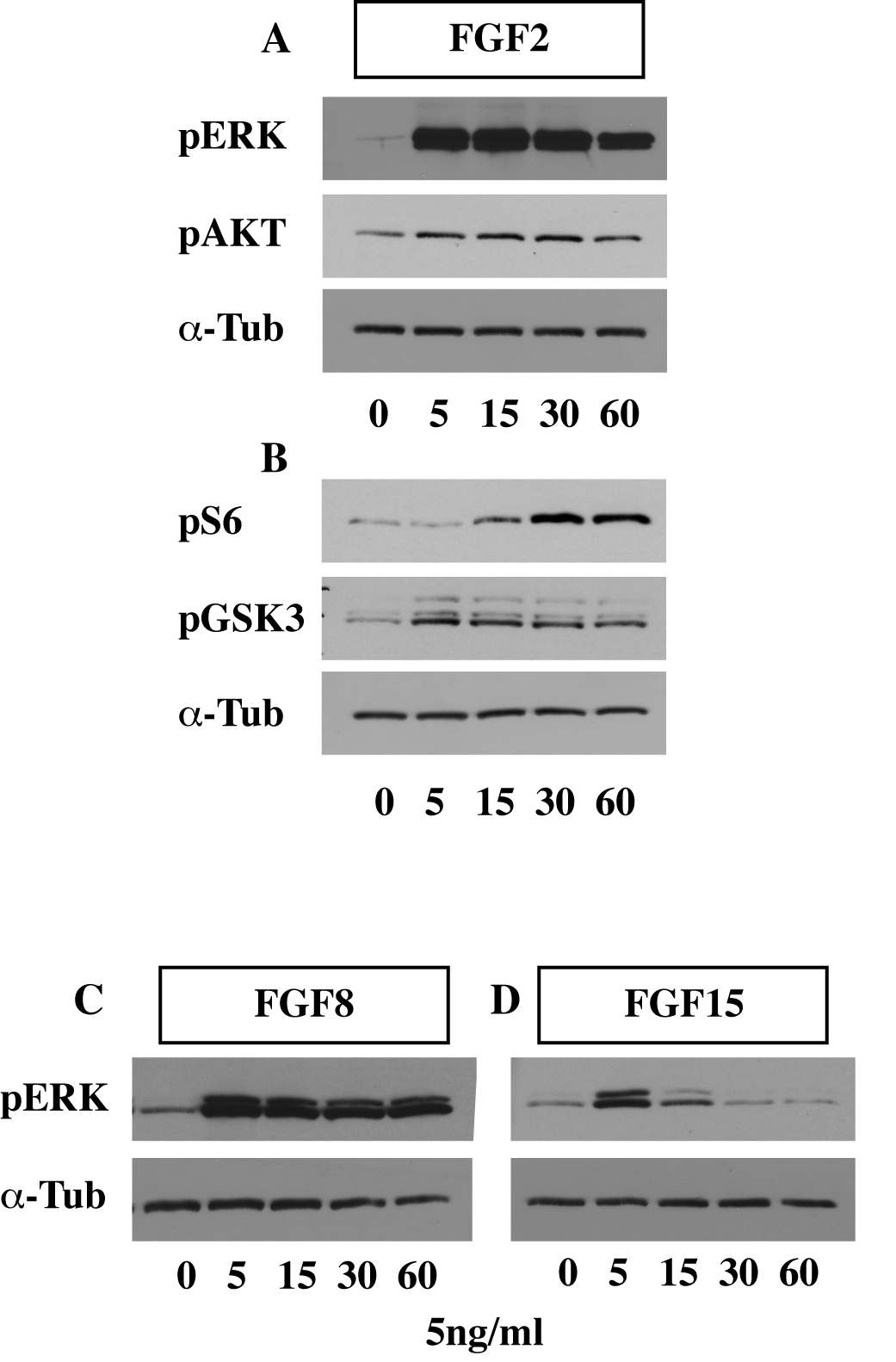

Supplement: Additional file 7 — Analysis of the downstream cytoplasmic effectors induced by FGF2. (a, b): comparison of phosphorylation of four proteins that are modified in response to FGF2. E12.5 primary cortical cultures were starved for 24 hours and treated with recombinant FGF2. Cell lysates were analyzed after 0, 5, 15, 30 and 60 minutes by immunoblotting to detect phosphorylated forms of pERK 42/44 (a, top panel), pAKT (a, middle panel), pS6 (b, top panel), and pGSK3 (b, middle panel). The α-Tubulin antibody (a, b, bottom panels) was used for normalization. Analysis of the phosphorylation of pERK (42/44) induced by FGF8 and FGF15 at 5 ng/ml (c and d, respectively, top panels). The α-Tubulin antibody was used for normalization (c, d, bottom panels). The numbers under the bands indicate the fold-induction, or reduction, with respect to T0. [file 1749-8104-3-17-S7.tiff]
